# Polymorphism of [Cu_15_(PhCH_2_CH_2_S)_13_(PPh_3_)_6_][BF_4_]_2_ and Double-Helical Assembly of [Cu_18_H(PhCH_2_CH_2_S)_14_(PPh_3_)_6_Cl_3_]: Origin of Two Chiral Nanoclusters with Triple-Helical Core from Intermediates

**DOI:** 10.1021/acsmaterialslett.4c02148

**Published:** 2025-01-02

**Authors:** Abhijit Nag, Abdul Mannan Butt, Moon Young Yang, Praveen B. Managutti, Bilal Masood Pirzada, M. Infas H. Mohideen, Ahmed L. Abdelhady, Mohamed Abu Haija, Sharmarke Mohamed, Boris V. Merinov, William A. Goddard, Ahsanulhaq Qurashi

**Affiliations:** † Department of Chemistry, Khalifa University of Science and Technology, Abu Dhabi 127788, United Arab Emirates; ‡ Center for Catalysis and Separations, Khalifa University of Science and Technology, Abu Dhabi 127788, United Arab Emirates; # Chemical Crystallography Laboratory, Khalifa University of Science and Technology, Abu Dhabi 127788, United Arab Emirates; § Materials and Process Simulation Center (MSC), California Institute of Technology, Pasadena, California 91125, United States

## Abstract

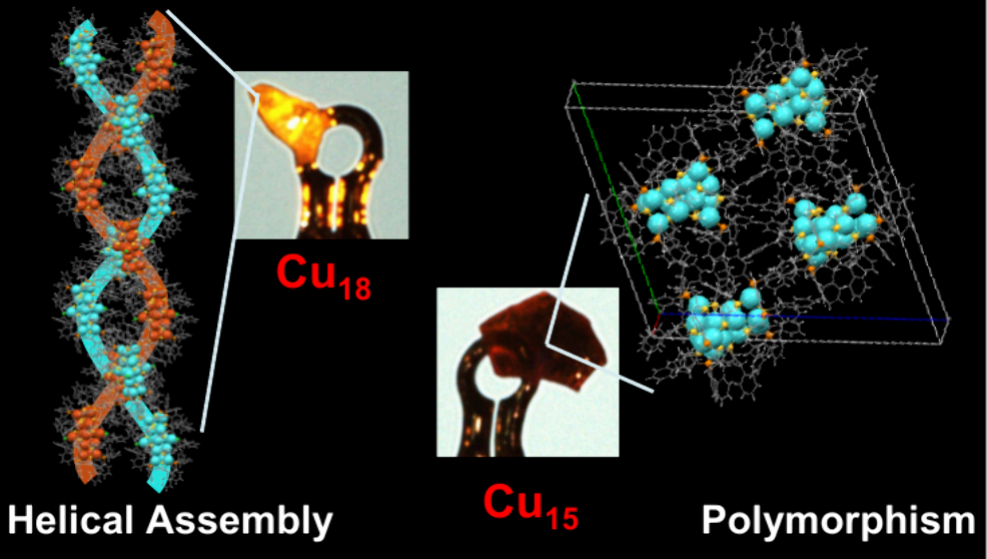

Here, we report the solvent-induced polymorphism in [Cu_15_(PET)_13_(TPP)_6_][BF_4_]_2_(Cu_15_) (TPP = triphenylphosphine, PET = 2-phenylethanthiol), and double-helical assembly of the [Cu_18_H(PET)_14_(TPP)_6_Cl_3_] (Cu_18_) nanocluster (NC) from reaction intermediates. Both copper NCs have an intrinsically chiral triple-stranded helicate metal core, unlike traditional copper NCs with a polyhedral-based kernel. The chiral structure of Cu_15_ resembles an enantiomeric pair in the unit cell. Moreover, Cu_18_ has a three-layered 3D chirality of a sandwich constructed of sulfur-bridged copper NCs aligned in a top-middle-down configuration. Furthermore, the Cu_18_ NC self-hierarchically assembles into a complex double-stranded helix secondary structure sustained by noncovalent interactions. Electrospray ionization mass spectrometry (ESI-MS), density functional theory (DFT), and X-ray photoelectron spectroscopy (XPS) were utilized to validate the single-crystal X-ray diffraction (SCXRD) data. Overall, this study provides an interesting example of polymorphism, chirality, and hierarchical double-helical assembly of NCs, allowing for extensive understanding of complicated structures at the atomic level.

Noble-metal nanoclusters (NMNCs) have attracted enormous interest for decades, due to their potential uses in various areas, including sensors, biological processes, optics, and catalysis.^1−9^ X-ray crystallography is used to determine the crystal structures of NCs created with high atomic precision.^10−13^ Recently, the research has focused on creating unique crystal structures for NCs to improve their various properties and applications.^14−24^ In this context, Cu NCs are potential candidates due to their unique structure and physical and chemical properties. As a result, the design, synthesis, and use of Cu NCs across various fields have become attractive.^25−34^

Single crystals, also known as nanoparticle super lattices, are formed by the self-organization of ligands on metal surfaces and their noncovalent interactions.^35−38^ For many NCs, secondary ligands also aid in cluster stabilization, even if the principal ligands of a particular cluster frequently shield the metal core.^10,39−42^ Different cluster assemblies are thus made feasible by intercluster interactions mediated by primary, secondary, or a combination of ligands. The crystallization of the well-known silver cluster Ag_44_(p-MBA)_30_Na_4_, often known as Ag_44_, is caused by hydrogen bonding (HB) of carboxylic acids.^37^ Similarly, NCs like Au_102_, Au_246_, Au_103_ exhibit comparable noncovalent interactions;^35,36,38^ the principal interactions of these NCs include C–H···π, HB, and van der Waals (vdW), depending on the ligands. Even though they are modest, these interactions could have significant electronic effects such as luminescence.^43^

Structural isomerism and polymorphism are common phenomena observed in metal NCs with the same chemical formula.^44^ The structure–property relationship can be better understood by examining the structural isomerism in gold and silver NCs, as these compounds have distinct properties. A few recent papers have focused on the structural isomers of NC due to the challenging nature of the synthesis and structural determination.^44^ The Au_38_T isomer, which has an Au_25_ inner core, exhibits far more catalytic activity in the hydrogenation of 4-nitrophenol, was discovered by Tian et al.^44^ Additionally, distinct symmetries and optical characteristics were displayed by Ag_29_ isomers doped with Pt.^45^ In the case of packing polymorphism, atomic packing modes are different with different unit cell parameters. Reports of polymorphism in NCs are still rare. The first instance of polymorphism (trigonal and cubic) in single crystals of Ag_29_(BDT)_12_(TPP)_4_^3–^ was reported by Nag et al.^43^ The C–H···π interactions of the secondary ligands (TPP) are more prominent in cubic phases, compared to trigonal phases, leading to a stronger stiffness of the structure and, consequently, a higher luminescence efficiency. Han et al. reported polymorphism of Cu_23_ NC, which crystallized in two polymorphs (*R*3*c* and *R*3̅), depending on the solvent used.^46^ Fu et al. reported the polymorphism of Au_19_Ag_4_(S-Adm)_15_, which showed *P*2_1_/*n*, *P*1̅, and *P*2_1_/*c* space groups, respectively.^47^ Polymorphism in Ni_6_ NC was also explored by Mandal et al.^48^

The creation of helical nanosized superstructures has long been challenging, and little has been accomplished in the field of NCs.^49^ The self-assembly of monomers is crucial for the fabrication of synthetic helical structures. The abundance of functional groups allows them to be used as building blocks for complex intermolecular aggregates.^18,50,51^ Metal NCs may be interesting candidates for monomers in helical self-assembled superstructures. In this context, Zhu et al. reported the triple-helical assembly of Au_6_Cu_6_ NCs.^52^ Later, Sun et al. studied the double-helical assembly of Cu_18_ NCs.^53^

Herein, we disclose the solvent-induced polymorphism in [Cu_15_(PET)_13_(TPP)_6_][BF_4_]_2_ (Cu_15_) (TPP = triphenylphosphine, PET = 2-phenylethanthiol), and double-helical assembly of [Cu_18_H(PET)_14_(TPP)_6_Cl_3_] (Cu_18_) from the reaction intermediate. Both copper NCs have an intrinsically chiral triple-stranded helicate metal core. The study reveals that Cu_15_ and Cu_18_ have distinct chiral structures, with Cu_15_ having enantiomeric pairs, while Cu_18_ has a complex double-stranded helix secondary structure. Electrospray ionization mass spectrometry (ESI-MS), density functional theory (DFT) calculations, and XPS were used to support the SCXRD data.

## Single-Crystal X-ray Diffraction (SCXRD) of [Cu_15_(PET)_13_(TPP)_6_][BF_4_]_2_

**Figure 1:**
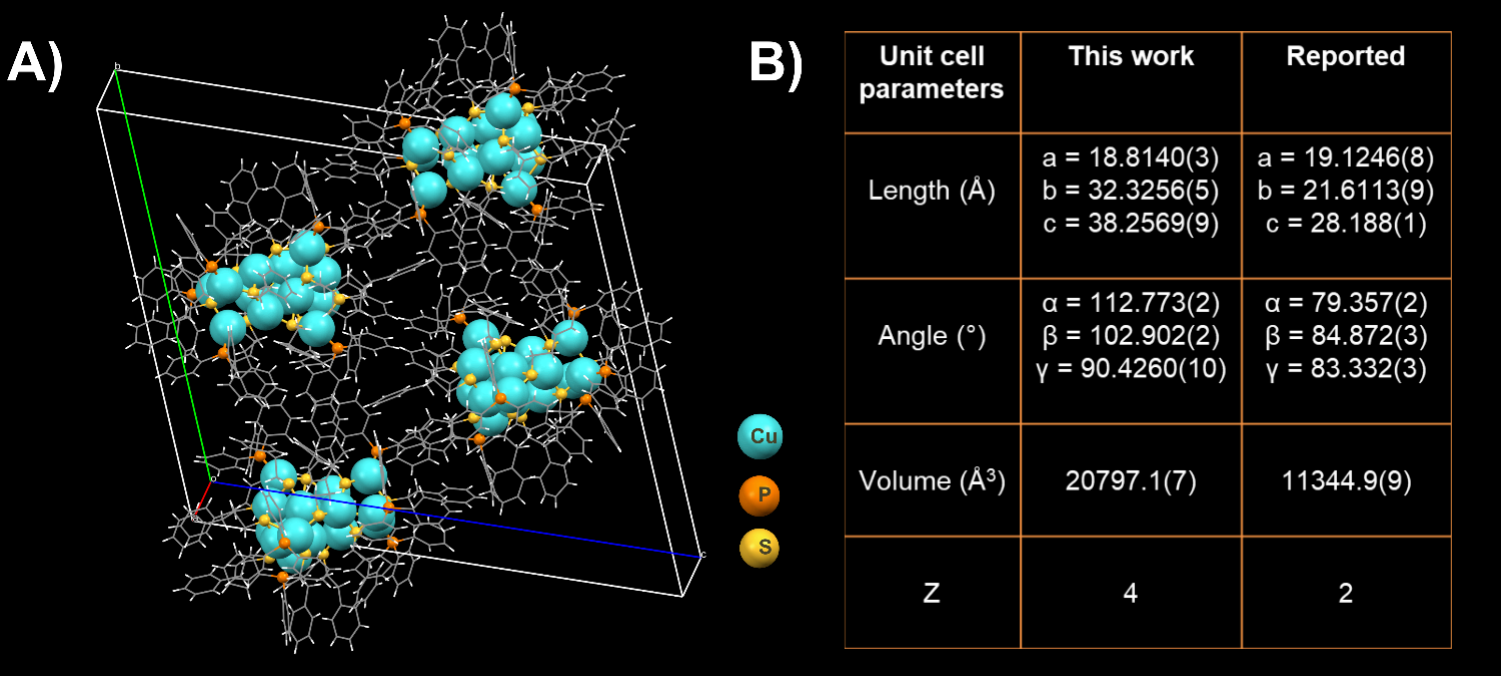
(A) Unit cell of the Cu_15_ NC in the present work. (B) Comparison of the unit cell parameters for the present work and the reported results.^54^

Bakr et al. disclosed the crystallization of Cu_15_ NC from the mixture of chloroform/hexane at 8 °C in the refrigerator after 2 days.^54^ We changed the crystallization method of the NC, which resulted in the formation of polymorphic crystals of the Cu_15_ NC (Figure S1). More details of the crystallization processes are given in the Supporting Information (SI). Three crystals of Cu_15_ NC were tested randomly, and we discovered that they are all NCs packed in the *P*1̅ space group with different unit-cell parameters with previously reported NC results (see Figures 1A and 1B, and Table S1). The Cu_15_ NC has two pairs of enantiomers per unit cell and produces a racemate. A similar polymorphism was observed for small organic molecules having the same space groups.^55^

**Figure 2:**
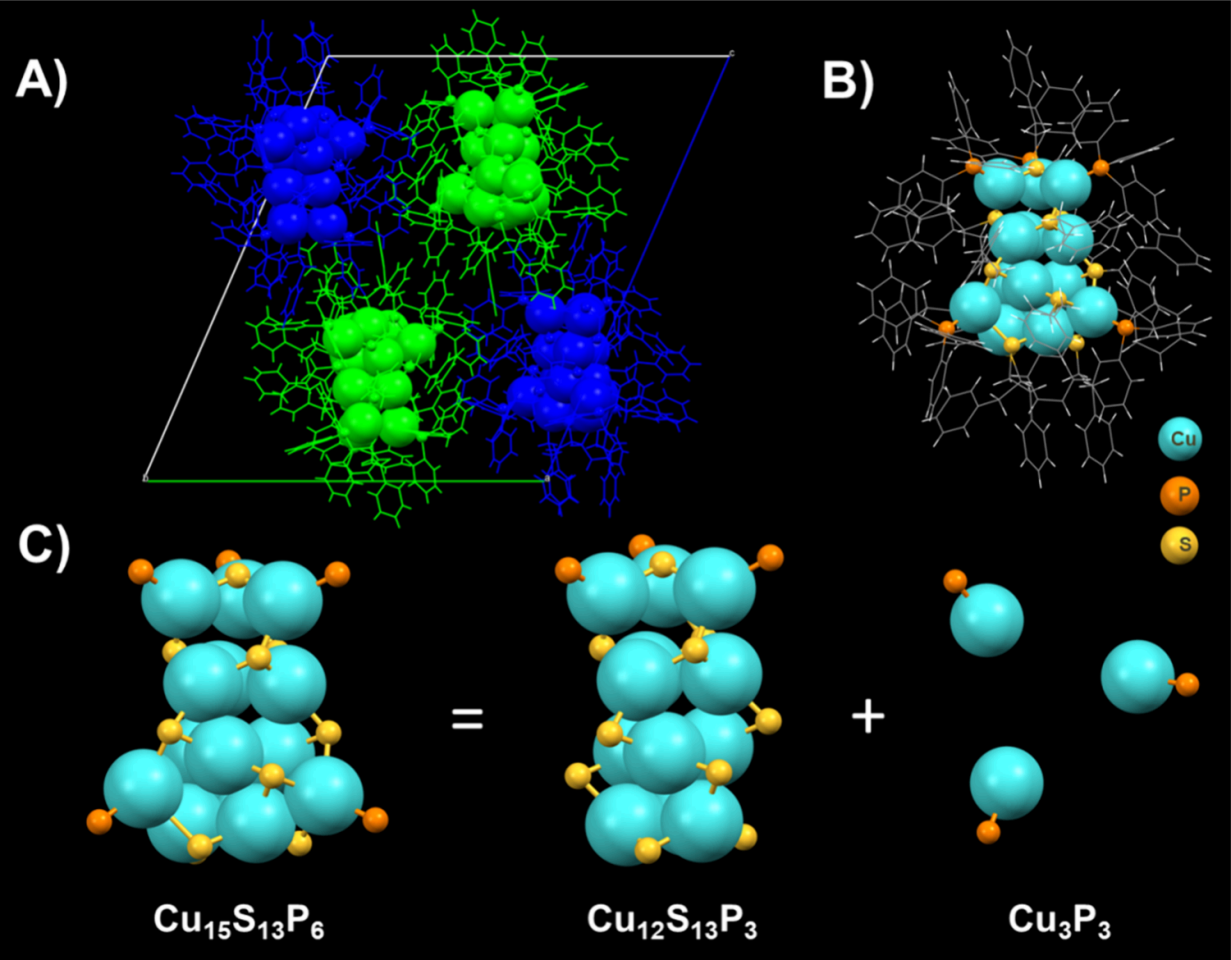
(A) Symmetry equivalence presentation of the Cu_15_ NC in the unit cell. (B) Crystal structure of Cu_15_ NC. (C) Structure anatomy of the Cu_15_ NC.

The unit cell consists of enantiomeric pairs of Cu_15_ NC with a 1:1 racemic mixture (Figure 2A).^54^ The position of the aromatic ligands on the surface of NC is significantly impacted by geometric restrictions. The surface structure is mostly controlled and stabilized by weak intermolecular ligand interactions, such as C–H···π (Figure S2). Therefore, the supramolecular dimer structure that the two opposing enantiomers adopted is caused by C–H···π interactions (Figure S2). These weak intercluster C–H···π and π–π interactions between benzene rings were previously observed in other NCs.^35−38,43^ The NC consists of 15 Cu atoms, 13 PET ligands, and 6 TPP secondary ligands (Figure 2B). A comprehensive molecular architecture examination reveals that the Cu_15_ NC has an intrinsically chiral triple-stranded helicate Cu_12_ core and Cu_3_ atoms (Figure 2C). The 12-atom metal core structure comprises three Cu_4_ strands, each as an arch-shaped array, yielding a unique metal aggregate-based triple helix with a [3 × 4] metal ion array. The Cu–Cu metal distances are similar to the previously reported structure.^54^ Among the 13 PET ligands, 12 PET ligands bind with the Cu atom via μ_3_-η^1^,η^1^,η^1^ mode, while the remaining PET present in the Cu_3_ unit binds via a μ_2_-η^1^,η^1^ mode. The Cu–S bond lengths range from 2.252 to 2.371 Å. Moreover, TPP ligands are connected by a single Cu–P bond to the Cu_15_ NC. Three TPP ligands out of the six occupy the top region of the Cu_15_ NC, while the remaining three are located in the lower section. In this way, they fulfill the steric and electronic surroundings of the Cu_15_ NC. The Cu–P bond lengths range from 2.210 Å to 2.231 Å. Furthermore, X-ray photoelectron spectroscopy (XPS) also revealed the Cu, P, S, and C elements in the Cu_15_ NC (Figure S3). Cu(I) was detected using Cu 2p_3/2_ at 930.90 eV and Cu 2p_1/2_ at 950.60 eV. Additionally, there is no satellite signal at 943 eV, indicating the lack of Cu(II) in Cu_15_. Furthermore, the Cu LMM Auger spectrum with a prominent peak at 570.78 eV reveals that all Cu atoms are in the Cu(I) oxidation state,^25^ which is consistent with the overall composition of Cu_15_ (Figure S4). ESI MS data also support the Cu(I) oxidation state the presence of other elements, which is presented later.

## SCXRD Data of [Cu_18_H(PET)_14_(TPP)_6_Cl_3_]

**Figure 3:**
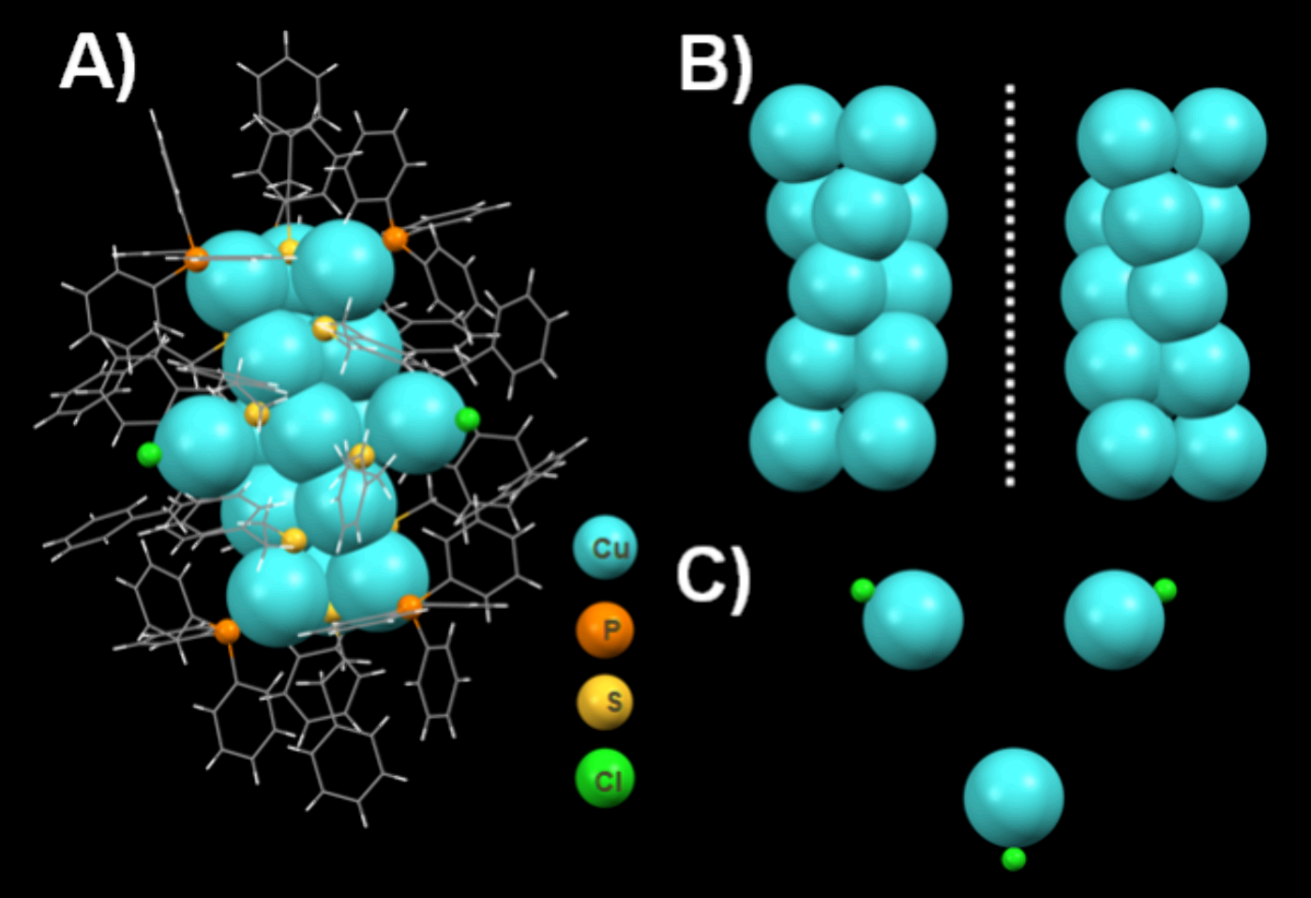
(A) Crystal structure of the Cu_18_ NC. (B) X-ray crystal structures of the enantiomeric pairs of the NC possessing a triple-helical Cu_15_ core. (C) A three Cu–Cl unit.

The precise composition of Cu_18_ was identified as [Cu_18_H(PET)_14_(TPP)_6_Cl_3_] using single-crystal X-ray diffraction (SCXRD) (Figure 3A) and various experimental and computational methods. More details about the cell parameters are provided in Table S2. Photographs of the crystals of the NC are provided in Figure S5. The unit-cell parameters, synthesis method, and ligand (Cl^–^) differ from the previously reported Cu_18_ crystal structure.^53^ A comparison of the unit-cell parameters between the previously reported Cu_18_ and the present work is provided in Table S3. The structural anatomy of Cu_18_ in the present work can be visualized as a conventional core–shell structure with a Cu_15_ metal kernel. The 15-atom metal core structure consists of three Cu_5_ strands, each as an arch-shaped array, yielding a unique metal aggregate-based triple helix with a [3 × 5] metal ion array. The triple-helical Cu_15_ core has approximate D_3_ symmetry with a pseudo C_3_ rotational axis passing through the helical axis and three pseudo C_2_ axis through the middle Cu_3_ triangle (Figures 3B and 3C). A protection shell encapsulates the Cu_15_ core, formed by 14 PETs, 6 TPPs, and 3 CuCl motifs. Furthermore, XPS revealed the existence of Cu, P, S, Cl, and C elements in the Cu_18_ NC (Figure S6). Cu(I) was detected using Cu 2p_3/2_ at 931.12 eV and Cu 2p_1/2_ at 950.90 eV. Additionally, there is no satellite signal at 943 eV, indicating the lack of Cu(II) in Cu_18_. Furthermore, the Cu LMM Auger spectrum with a prominent peak at 570.75 eV reveals that all Cu atoms are in the Cu(I) oxidation state,^25^ which agrees with the overall composition of Cu_18_ (Figure S7). ESI MS data also support the Cu(I) oxidation state and the presence of other elements, which is presented later.

**Figure 4:**
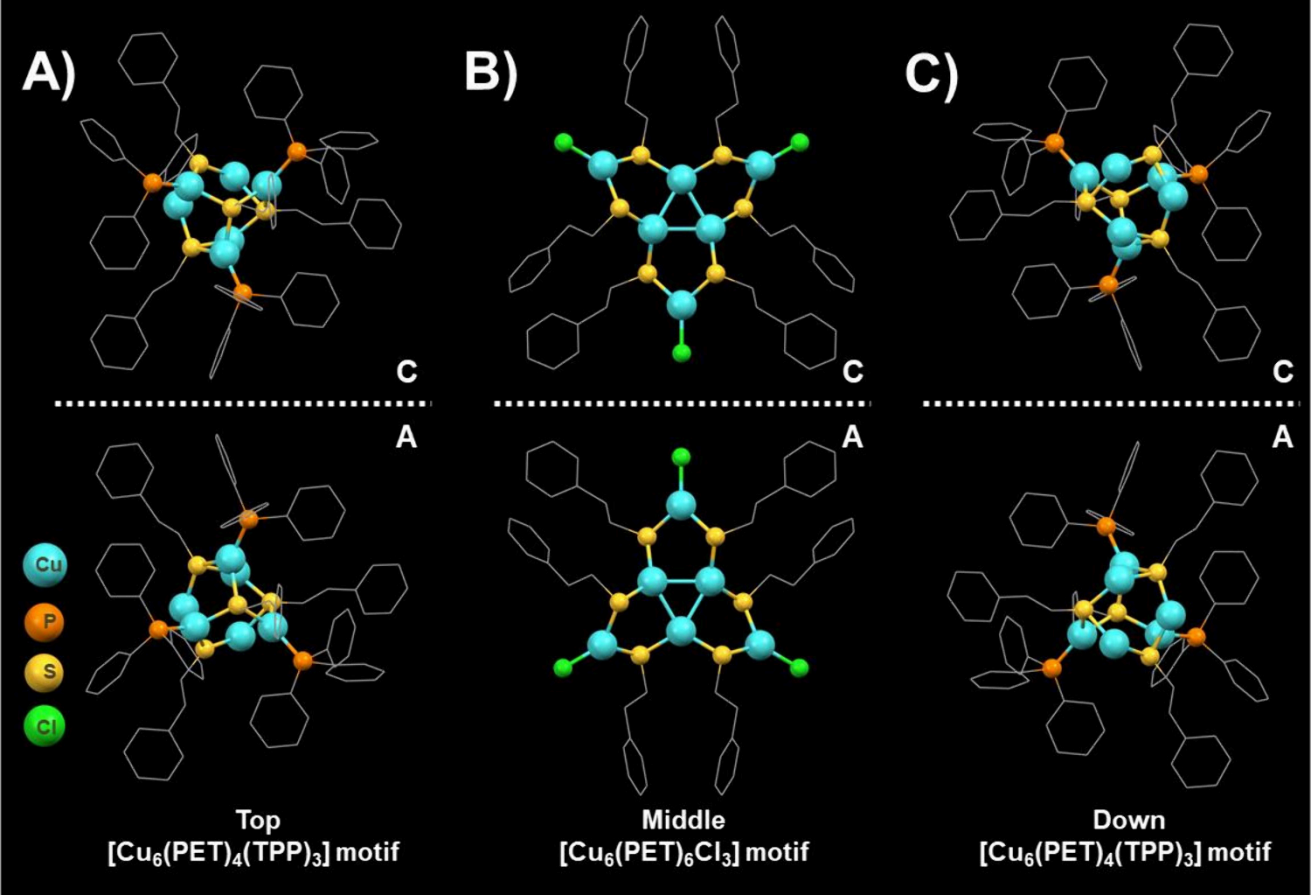
Three chiral layers ((A) top, (B) middle, and (C) down) are parallelly aligned, resulting in the unique 3D chirality of Cu_18_ NC. Mirror images of each layer are presented. In the figure, C = clockwise and A = anticlockwise.

Cu_18_ NC has a unique 3D chirality, consisting of three layers along the quasi-3-fold axis of symmetry: top, middle, and bottom. The upper and bottom layers contain a hexanuclear motif [Cu_6_(PET)_4_(TPP)_3_], which has a distorted Cu_6_S_4_ adamantane-like framework with Cu–S distances of 2.239–2.272 Å, well within the range normally associated with copper thiolates (Figures 4A and 4C). There is no hint of Cu···Cu interactions within the Cu_6_S_4_ cage. The central layer is a [Cu_6_(PET)_6_Cl_3_] motif with an isosceles triangular Cu_3_ plane with an average Cu–Cu distance of 2.673 Å, suggesting cuprophilic interactions (Figure 4B). The Cu_3_ plane is coordinated by three bidentate [Cu(PET)_2_Cl] metalloligands via Cu–S bonds to form three Cu_3_S_2_ five-membered rings. The three chiral layers are aligned parallel in a “top-middle-down” fashion and joined through sulfur-bridging Cu–S bonds, resulting in the unique 3D chirality of Cu_18_ NC. Cu_18_ possesses a distinct 3D chirality because of its three chiral layers’ parallel alignment in a “top-middle-down” pattern and the sulfur-bridging Cu–S bonds that unite them. Theoretically, assembling the three chiral layers can produce four possible stereoisomers: enantiomers CCC-Cu_18_ and AAA-Cu_18_, as well as enantiomers CAC-Cu_18_ and ACA-Cu_18_. Because of the steric repulsion of protective ligands, the copper-thiolate NCs in the upper and lower layers always adopt the same spiral orientations and are identical with the core layer, which is the remarkable property that gives rise to this 3D chirality. As a result, the crystal only shows the two chiral-specific enantiomers of CCC-Cu_18_ and AAA-Cu_18_ (Figure 4). Notably, multilayered 3D chirality is present in a variety of biological macromolecules, including DNA and proteins. Similarly, chirally organized multilayered structures are found in protein folding. This thus provides an interesting example of NCs that could mimic the existing cluster-based, multiple-layered 3D chirality in Cu_18_.

**Figure 5:**
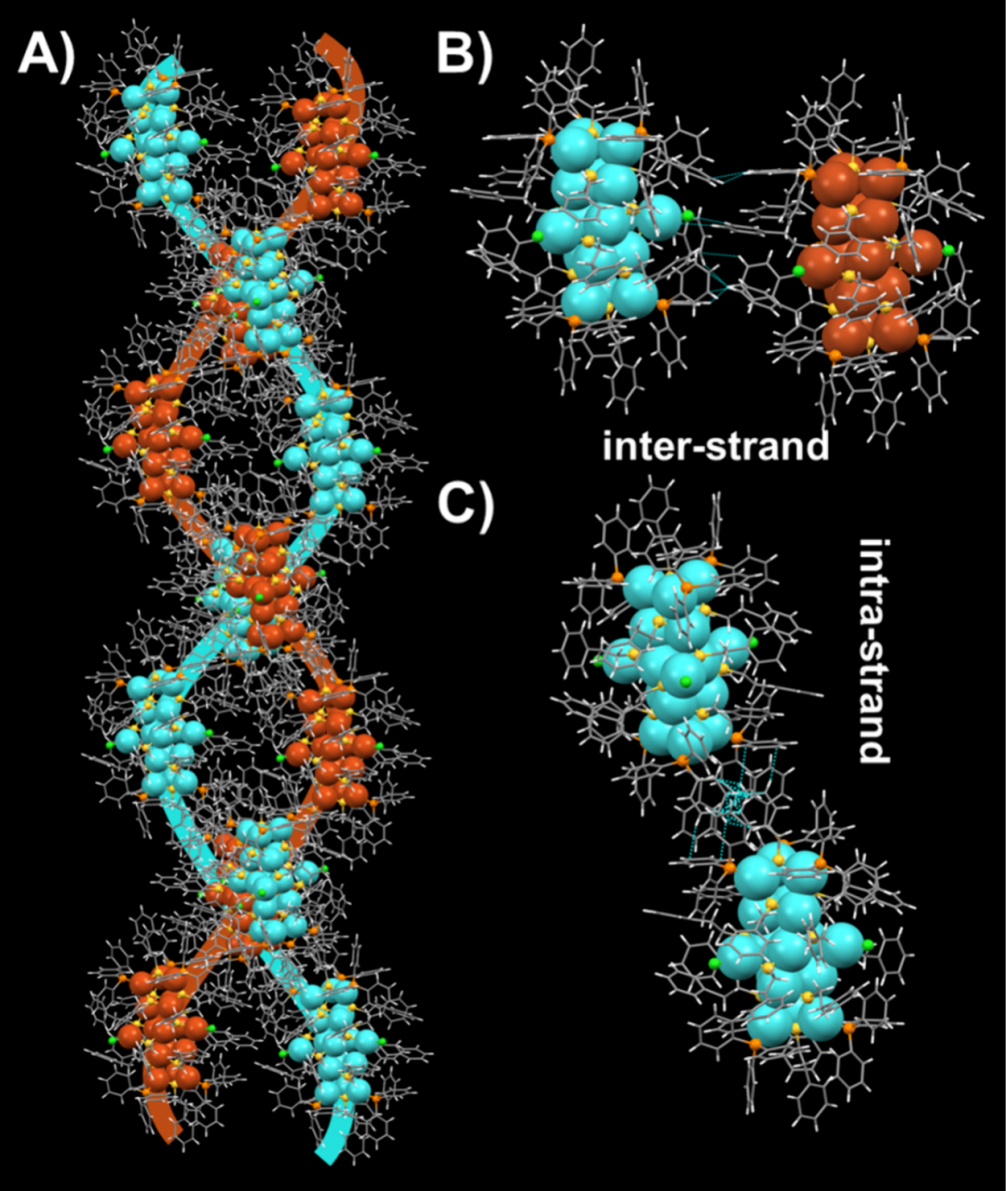
(A) Intercluster helical chains in the super crystal lattice. (B) Interstrand and (C) intrastrand noncovalent interactions present in the helix structure.

Another noteworthy structural property of the Cu_18_ NC is that the NCs form intercluster helical chains in the super crystal lattice when the unit cell is extended along the *Z*-direction. Figure 5A shows how the spirals interlock to form two strands of a double helix. According to these findings, cluster–cluster interactions in the intrastrand and interstrand are closely related to the structure of protective ligands and may play a key role in the stabilization of NC secondary structures (Figures 5B and 5C). In the interstrand, the presence of C–H···π, C–H···Cl, and C–H···H–C interactions was observed, with average distances in the range of 2.720–2.890 Å, 2.910 Å, and 2.310–2.361 Å, respectively. In the intrastrand, the presence of C–H···π interactions with an average distance of 2.469 Å was observed. The presence of weak noncovalent interactions is mainly responsible for this double helix structure. It is worth mentioning that the double-helical assembly of Cu_18_ NC is similar to the well-known double helix structure of DNA but not identical. The DNA double helix is made up of two helical polynucleotide chains with sugar–phosphate backbones that are covalently attached to each other. The two chains are connected by many hydrogen bonds. In our scenario, however, the chiral Cu_18_ NCs form a supramolecular double-stranded helix that is sustained by both intrastrand and interstrand noncovalent interactions.

## ESI-MS Study of the Reaction Intermediate and DFT Optimization

To verify the final composition of Cu_15_ and Cu_18_ NC from the reaction intermediate, ESI-MS was employed after dissolving the precipitate obtained from the reaction after 30 min (see more details in the SI) in dichloromethane (DCM). Figure S8 shows the ESI-MS spectrum of Cu_15_ and Cu_18_ NC in positive mode. The peak [Cu_15_(PET)_13_(TPP)_6_]^2+^ corresponds to *m*/*z* = 2155. The ascribed composition is confirmed by precisely matching the calculated and observed isotope patterns (inset of Figure S8). The characteristic difference (Δ*m*/*z* = 0.5) between two successive peaks of the isotopic distribution indicates that the NC is divalent. Furthermore, the presence of counterions was verified by negative mode ESI-MS. At *m*/*z* = 87, a single charged signal was seen, corresponding to BF_4_^–^ (Figure S9). Most significantly, these experimental findings unambiguously imply that the formula of the synthesized NMNC is [Cu_15_(PET)_13_(TPP)_6_][BF_4_]_2_. This is corroborated by the free valence electron count rule, which shows that this NC has no free electrons. The composition of the Cu_18_ NC was also further determined by electrospray ionization mass spectrometry (ESI-MS) analysis. However, no parent cluster ion signal was identified in either positive or negative ionization modes, which showed that Cu_18_ is neutral. Cu_18_ may be a charge neutral cluster, as evidenced by the lack of counterions in the crystal structure. Alternatively, to ensure electroneutrality of [Cu_18_(PET)_14_(TPP)_6_Cl_3_]^+^ under NaBH_4_ reduction conditions, one H^–^ ligand is likely required. MS peaks were found in the mass range of *m*/*z* 2200–2400 (Figure S8). These peaks were attributed to [Cu_18_H(PET)_14_Cl_2_(TPP)_6_ + Cu(I)]^2+^, [Cu_18_H(PET)_14_Cl(TPP)_6_]^2+^, [Cu_17_(PET)_14_Cl(TPP)_6_]^2+^, [Cu_17_H(PET)_14_(TPP)_6_]^2+^, [Cu_18_H(PET)_14_Cl_2_(TPP)_5_ + Cu(I)]^2+^, and [Cu_18_H(PET)_14_Cl_2_(TPP)_4_ + Cu(I)]^2+^. They become ionized by adding a Cu(I) ion or losing chlorides or hydride ions. The charge balance calculations also support the presence of hydride in the NC. The ascribed composition is confirmed by precisely matching of the calculated and observed isotope patterns (Figure S8). The characteristic difference (Δ*m*/*z* = 0.5) between two successive peaks of the isotopic distribution indicates that the NC is divalent. In order to prove the existence of the hydrides, a deuterated cluster was created by substituting NaBD_4_ for NaBH_4_ as the reducing agent. In fact, Figure S10 displays a mass spectrum that is upshifted by *m*/*z* 0.5 from Cu_18_, indicating a greater mass of 1. Most significantly, these experimental findings unambiguously imply that the formula of the synthesized NC is [Cu_18_H(PET)_14_(TPP)_6_Cl_3_]. This is also corroborated by the free valence electron count rule, which shows that the NC has no free electrons. ESI–MS study confirmed the formation of both the Cu_15_ and Cu_18_ NCs in the reaction intermediate. To understand the overall experimental results, we note that both Cu_15_ and Cu_18_ NC have similar chiral triple-stranded helicate metal cores (Figure S11) that crystallize with enantiomeric pairs. This suggests that both NCs have similar growth mechanisms. This also supports the formation of the two NCs in the same reaction. If the reaction were continued for a longer time (5 h), then only Cu_15_ NC was formed.^54^

To determine the hydride location in the Cu_18_ NC, we performed DFT calculations. Based on the previous report,^53^ we examined both the center of the Cu_3_ plane (planar) and a position out of the Cu_3_ plane (pyramid) (Figure S12). After geometry optimization, the hydride in both models ended up in nearly the same position, slightly out of plane by ∼0.7 Å (Figure S12). The average distance between the hydride and the three nearest Cu atoms was 1.74 Å, which is consistent with previously reported trinuclear Cu hydride systems.^51−54^

In summary, we discussed the solvent-induced polymorphism in [Cu_15_(PET)_13_(TPP)_6_][BF_4_]_2_, and the double-helical assembly of [Cu_18_H(PET)_14_(TPP)_6_Cl_3_] generated from the reaction intermediate. This study revealed that both the copper NCs have an intrinsically chiral triple-stranded helicate metal core. The chiral structure of Cu_15_ assembles with the enantiomers in the unit cell. Moreover, Cu_18_ NC exhibits a unique 3D chirality and a secondary double-helical structure resembling DNA. Electrospray ionization mass spectrometry (ESI-MS), density functional theory (DFT) calculations, and XPS were used to support the SCXRD data. This study highlights the intricate and smart self-assembly of copper nanoclusters, similar to that of biological molecules like DNA. It also provides an intriguing model for studying the relationship between the reaction intermediate and structural properties of NCs.

## Supporting Information Available

CIF data for Cu_18_ (PDF)
CIF data for Cu_15_ (PDF)
Crystallographic structure of the Cu_18_ nanocluster (CIF)
Crystallographic structure of the Cu_15_ nanocluster (CIF)
Three-dimensional image of the Cu_15_ nanocluster (MP4)
Details regarding synthesis, instrumentation and characterization, including ESI-MS, X-ray crystallography, XPS, and DFT calculations; Figures S1–S12 and Tables S1–S3 (PDF)

## Supplementary Material

tz4c02148_si_002.pdf

tz4c02148_si_003.cif

tz4c02148_si_004.cif
