# Supplementary material for: Polymorphism of [Cu15(PhCH2CH2S)13(PPh3)6][BF4]2 and Double-Helical Assembly of [Cu18H(PhCH2CH2S)14(PPh3)6Cl3]: Origin of Two Chiral Nanoclusters with Triple-Helical Core from Intermediates
Source: ACS Mater Lett. 2025 Jan 2;7(2):442–9. doi: 10.1021/acsmaterialslett.4c02148 (PMC11795624; doi:10.1021/acsmaterialslett.4c02148)

checkCIF/PLATON report

You have not supplied any structure factors. As a result the full set of tests cannot be run.

THIS REPORT IS FOR GUIDANCE ONLY. IF USED AS PART OF A REVIEW PROCEDURE FOR PUBLICATION, IT SHOULD NOT REPLACE THE EXPERTISE OF AN EXPERIENCED CRYSTALLOGRAPHIC REFEREE.

No syntax errors found.   CIF dictionary   Interpreting this report

Datablock: cu2202

```
Bond precision:   C-C = 0.0175 A            Wavelength=1.54184

Cell:              a=25.9002(3)     b=27.6131(7)       c=32.6918(10)
                   alpha=90         beta=97.250(2)     gamma=90
Temperature:       100 K
```

	Calculated	Reported
Volume	23193.8(10)	23193.7(10)
Space group	C 2/c	C 1 2/c 1
Hall group	-C 2yc	-C 2yc
Moiety formula	C220 H216 Cl3 Cu18 P6 S14 [+ solvent]	C220 H216 Cl3 Cu18 P6 S14, 3[C2H3N.CH4O.H2O]
Sum formula	C220 H216 Cl3 Cu18 P6 S14 [+ solvent]	C229 H243 Cl3 Cu18 N3 O6 P6 S14
Mr	4744.85	5017.98
Dx,g cm-3	1.359	1.437
Z	4	4
Mu (mm-1)	3.966	4.021
F000	9692.0	10292.0
F000'	9595.33	
h,k,lmax	30,32,38	30,32,38
Nref	20490	20281
Tmin,Tmax	0.639,0.716	0.781,1.000
Tmin'	0.481	

```
Correction method= # Reported T Limits: Tmin=0.781 Tmax=1.000
AbsCorr = MULTI-SCAN

Data completeness= 0.990            Theta(max)= 66.589
```

R(reflections)= 0.1063(9251) wR2(reflections)= 0.3781(20281)

S = 1.176 Npar= 985

The following ALERTS were generated. Each ALERT has the format
test-name_ALERT_alert-type_alert-level.
Click on the hyperlinks for more details of the test.

Alert level A

PLAT411_ALERT_2_A Short Inter H...H Contact H11 ..H89 . 1.52 Ang.
3/2-x,3/2-y,1-z = 7_666 Check

Author Response: This alert is caused by severe disorder of the surface ligands.

Alert level B

PLAT084_ALERT_3_B High wR2 Value (i.e. > 0.25) .... 0.38 Report

Author Response: This alert is caused by week diffraction of the crystal.

PLAT234_ALERT_4_B Large Hirshfeld Difference S6 --C104 . 0.27 Ang.

Author Response: DELU restrains were applied to disorder of the surface ligands. However this does not indicate incorrect atom type assignment.

PLAT234_ALERT_4_B Large Hirshfeld Difference C7 --C55 . 0.30 Ang.

Author Response: DELU restrains were applied to disorder of the surface ligands. However this does not indicate incorrect atom type assignment.

PLAT234_ALERT_4_B Large Hirshfeld Difference C16 --C18 . 0.28 Ang.

Author Response: DELU restrains were applied to disorder of the surface ligands. However this does not indicate incorrect atom type assignment.

PLAT234_ALERT_4_B Large Hirshfeld Difference C64 --C74 . 0.26 Ang.

Author Response: DELU restrains were applied to disorder of the surface ligands. However this does not indicate incorrect atom type assignment.

PLAT241_ALERT_2_B High 'MainMol' Ueq as Compared to Neighbors of C82 Check

Author Response: This alert is caused by severe disorder of the surface ligands.

```
PLAT241_ALERT_2_B High   'MainMol' Ueq as Compared to Neighbors of        C97 Check
```

Author Response: This alert is caused by severe disorder of the surface ligands.

```
PLAT242_ALERT_2_B Low    'MainMol' Ueq as Compared to Neighbors of       Cu10 Check
```

Author Response: This alert is caused by severe disorder of the surface ligands.

```
PLAT341_ALERT_3_B Low Bond Precision on  C-C Bonds ...............    0.01746 Ang.
```

Author Response: This alert is caused by severe disorder of the surface ligands.

```
PLAT360_ALERT_2_B Short  C(sp3)-C(sp3) Bond  C32      - C106     .       1.31 Ang.
```

Author Response: This alert is caused by severe disorder of the surface ligands.

```
PLAT360_ALERT_2_B Short  C(sp3)-C(sp3) Bond  C50      - C90      .       1.25 Ang.
```

Author Response: This alert is caused by severe disorder of the surface ligands.

```
PLAT411_ALERT_2_B Short Inter H...H Contact  H89      ..H89      .       1.89 Ang.
                                           3/2-x,3/2-y,1-z  =      7_666 Check
```

Author Response: This alert is caused by severe disorder of the surface ligands.

Alert level C

```
PLAT026_ALERT_3_C Ratio Observed / Unique Reflections (too) Low ..        46% Check
PLAT082_ALERT_2_C High R1 Value .................................       0.11 Report
PLAT234_ALERT_4_C Large Hirshfeld Difference P1       --C31      .       0.20 Ang.
```

Author Response: DELU restrains were applied to disorder of the surface ligands. However this does not indicate incorrect atom type assignment.

```
PLAT234_ALERT_4_C Large Hirshfeld Difference P2       --C49      .       0.18 Ang.
```

Author Response: DELU restrains were applied to disorder of the surface ligands. However this does not indicate incorrect atom type assignment.

```
PLAT234_ALERT_4_C Large Hirshfeld Difference P2       --C85      .       0.18 Ang.
```

Author Response: DELU restrains were applied to disorder of the surface ligands. However this does not indicate incorrect atom type assignment.

PLAT234_ALERT_4_C Large Hirshfeld Difference P3 --C3 . 0.20 Ang.

Author Response: DELU restrains were applied to disorder of the surface ligands. However this does not indicate incorrect atom type assignment.

PLAT234_ALERT_4_C Large Hirshfeld Difference P3 --C64 . 0.25 Ang.

Author Response: DELU restrains were applied to disorder of the surface ligands. However this does not indicate incorrect atom type assignment.

PLAT234_ALERT_4_C Large Hirshfeld Difference P3 --C79 . 0.18 Ang.

Author Response: DELU restrains were applied to disorder of the surface ligands. However this does not indicate incorrect atom type assignment.

PLAT234_ALERT_4_C Large Hirshfeld Difference C14 --C24 . 0.25 Ang.

Author Response: DELU restrains were applied to disorder of the surface ligands. However this does not indicate incorrect atom type assignment.

PLAT234_ALERT_4_C Large Hirshfeld Difference C14 --C50 . 0.24 Ang.

Author Response: DELU restrains were applied to disorder of the surface ligands. However this does not indicate incorrect atom type assignment.

PLAT234_ALERT_4_C Large Hirshfeld Difference C29 --C57 . 0.24 Ang.

Author Response: DELU restrains were applied to disorder of the surface ligands. However this does not indicate incorrect atom type assignment.

PLAT234_ALERT_4_C Large Hirshfeld Difference C32 --C106 . 0.24 Ang.

Author Response: DELU restrains were applied to disorder of the surface ligands. However this does not indicate incorrect atom type assignment.

PLAT234_ALERT_4_C Large Hirshfeld Difference C34 --C44 . 0.18 Ang.

Author Response: DELU restrains were applied to disorder of the surface ligands. However this does not indicate incorrect atom type assignment.

PLAT234_ALERT_4_C Large Hirshfeld Difference C42 --C44 . 0.19 Ang.

Author Response: DELU restrains were applied to disorder of the surface ligands. However this does not indicate incorrect atom type assignment.

PLAT234_ALERT_4_C Large Hirshfeld Difference C64 --C66 . 0.22 Ang.

Author Response: DELU restrains were applied to disorder of the surface ligands. However this does not indicate incorrect atom type assignment.

PLAT234_ALERT_4_C Large Hirshfeld Difference C78 --C88 . 0.21 Ang.

Author Response: DELU restrains were applied to disorder of the surface ligands. However this does not indicate incorrect atom type assignment.

PLAT241_ALERT_2_C High 'MainMol' Ueq as Compared to Neighbors of C3 Check

Author Response: This alert is caused by severe disorder of the surface ligands.

PLAT241_ALERT_2_C High 'MainMol' Ueq as Compared to Neighbors of C6 Check

Author Response: This alert is caused by severe disorder of the surface ligands.

PLAT241_ALERT_2_C High 'MainMol' Ueq as Compared to Neighbors of C7 Check

Author Response: This alert is caused by severe disorder of the surface ligands.

PLAT241_ALERT_2_C High 'MainMol' Ueq as Compared to Neighbors of C8 Check

Author Response: This alert is caused by severe disorder of the surface ligands.

PLAT241_ALERT_2_C High 'MainMol' Ueq as Compared to Neighbors of C10 Check

Author Response: This alert is caused by severe disorder of the surface ligands.

PLAT241_ALERT_2_C High 'MainMol' Ueq as Compared to Neighbors of C13 Check

Author Response: This alert is caused by severe disorder of the surface ligands.

PLAT241_ALERT_2_C High 'MainMol' Ueq as Compared to Neighbors of C18 Check

Author Response: This alert is caused by severe disorder of the surface ligands.

PLAT241_ALERT_2_C High 'MainMol' Ueq as Compared to Neighbors of C31 Check

Author Response: This alert is caused by severe disorder of the surface ligands.

PLAT241_ALERT_2_C High 'MainMol' Ueq as Compared to Neighbors of C36 Check

Author Response: This alert is caused by severe disorder of the surface ligands.

PLAT241_ALERT_2_C High 'MainMol' Ueq as Compared to Neighbors of C38 Check

Author Response: This alert is caused by severe disorder of the surface ligands.

PLAT241_ALERT_2_C High 'MainMol' Ueq as Compared to Neighbors of C49 Check

Author Response: This alert is caused by severe disorder of the surface ligands.

PLAT241_ALERT_2_C High 'MainMol' Ueq as Compared to Neighbors of C50 Check

Author Response: This alert is caused by severe disorder of the surface ligands.

PLAT241_ALERT_2_C High 'MainMol' Ueq as Compared to Neighbors of C52 Check

Author Response: This alert is caused by severe disorder of the surface ligands.

PLAT241_ALERT_2_C High 'MainMol' Ueq as Compared to Neighbors of C55 Check

Author Response: This alert is caused by severe disorder of the surface ligands.

PLAT241_ALERT_2_C High 'MainMol' Ueq as Compared to Neighbors of C64 Check

Author Response: This alert is caused by severe disorder of the surface ligands.

PLAT241_ALERT_2_C High 'MainMol' Ueq as Compared to Neighbors of C72 Check

Author Response: This alert is caused by severe disorder of the surface ligands.

PLAT241_ALERT_2_C High 'MainMol' Ueq as Compared to Neighbors of C79 Check

Author Response: This alert is caused by severe disorder of the surface ligands.

```
PLAT241_ALERT_2_C High   'MainMol' Ueq as Compared to Neighbors of        C85 Check
```

Author Response: This alert is caused by severe disorder of the surface ligands.

```
PLAT241_ALERT_2_C High   'MainMol' Ueq as Compared to Neighbors of        C90 Check
```

Author Response: This alert is caused by severe disorder of the surface ligands.

```
PLAT241_ALERT_2_C High   'MainMol' Ueq as Compared to Neighbors of       C102 Check
```

Author Response: This alert is caused by severe disorder of the surface ligands.

```
PLAT241_ALERT_2_C High   'MainMol' Ueq as Compared to Neighbors of       C104 Check
```

Author Response: This alert is caused by severe disorder of the surface ligands.

```
PLAT241_ALERT_2_C High   'MainMol' Ueq as Compared to Neighbors of       C106 Check
```

Author Response: This alert is caused by severe disorder of the surface ligands.

```
PLAT242_ALERT_2_C Low    'MainMol' Ueq as Compared to Neighbors of        Cu4 Check
```

Author Response: This alert is caused by severe disorder of the surface ligands.

```
PLAT242_ALERT_2_C Low    'MainMol' Ueq as Compared to Neighbors of         S6 Check
```

Author Response: This alert is caused by severe disorder of the surface ligands.

```
PLAT242_ALERT_2_C Low    'MainMol' Ueq as Compared to Neighbors of         P1 Check
```

Author Response: This alert is caused by severe disorder of the surface ligands.

```
PLAT242_ALERT_2_C Low    'MainMol' Ueq as Compared to Neighbors of         P2 Check
```

Author Response: This alert is caused by severe disorder of the surface ligands.

```
PLAT242_ALERT_2_C Low    'MainMol' Ueq as Compared to Neighbors of         P3 Check
```

Author Response: This alert is caused by severe disorder of the surface ligands.

```
PLAT242_ALERT_2_C Low    'MainMol' Ueq as Compared to Neighbors of         C1 Check
```

Author Response: This alert is caused by severe disorder of the surface ligands.

```
PLAT242_ALERT_2_C Low    'MainMol' Ueq as Compared to Neighbors of         C4 Check
```

Author Response: This alert is caused by severe disorder of the surface ligands.

```
PLAT242_ALERT_2_C Low    'MainMol' Ueq as Compared to Neighbors of        C14 Check
```

Author Response: This alert is caused by severe disorder of the surface ligands.

```
PLAT242_ALERT_2_C Low    'MainMol' Ueq as Compared to Neighbors of        C16 Check
```

Author Response: This alert is caused by severe disorder of the surface ligands.

```
PLAT242_ALERT_2_C Low    'MainMol' Ueq as Compared to Neighbors of        C28 Check
```

Author Response: This alert is caused by severe disorder of the surface ligands.

```
PLAT242_ALERT_2_C Low    'MainMol' Ueq as Compared to Neighbors of        C29 Check
```

Author Response: This alert is caused by severe disorder of the surface ligands.

```
PLAT242_ALERT_2_C Low    'MainMol' Ueq as Compared to Neighbors of        C30 Check
```

Author Response: This alert is caused by severe disorder of the surface ligands.

```
PLAT242_ALERT_2_C Low    'MainMol' Ueq as Compared to Neighbors of        C32 Check
```

Author Response: This alert is caused by severe disorder of the surface ligands.

```
PLAT242_ALERT_2_C Low    'MainMol' Ueq as Compared to Neighbors of        C34 Check
```

Author Response: This alert is caused by severe disorder of the surface ligands.

```
PLAT242_ALERT_2_C Low    'MainMol' Ueq as Compared to Neighbors of        C66 Check
```

Author Response: This alert is caused by severe disorder of the surface ligands.

```
PLAT242_ALERT_2_C Low    'MainMol' Ueq as Compared to Neighbors of        C76 Check
```

Author Response: This alert is caused by severe disorder of the surface ligands.

```
PLAT242_ALERT_2_C Low    'MainMol' Ueq as Compared to Neighbors of        C84 Check
```

Author Response: This alert is caused by severe disorder of the surface ligands.

```
PLAT242_ALERT_2_C Low    'MainMol' Ueq as Compared to Neighbors of        C88 Check
```

Author Response: This alert is caused by severe disorder of the surface ligands.

```
PLAT242_ALERT_2_C Low    'MainMol' Ueq as Compared to Neighbors of       C108 Check
```

Author Response: This alert is caused by severe disorder of the surface ligands.

```
PLAT242_ALERT_2_C Low    'MainMol' Ueq as Compared to Neighbors of       C110 Check
```

Author Response: This alert is caused by severe disorder of the surface ligands.

```
PLAT260_ALERT_2_C Large Average Ueq of Residue Including       Cu1      0.204 Check
PLAT360_ALERT_2_C Short  C(sp3)-C(sp3) Bond  C15      - C104     .       1.37 Ang.
```

Author Response: This alert is caused by severe disorder of the surface ligands.

```
PLAT360_ALERT_2_C Short  C(sp3)-C(sp3) Bond  C26      - C48      .       1.42 Ang.
```

Author Response: This alert is caused by severe disorder of the surface ligands.

```
PLAT360_ALERT_2_C Short  C(sp3)-C(sp3) Bond  C30      - C46      .       1.42 Ang.
```

Author Response: This alert is caused by severe disorder of the surface ligands.

```
PLAT360_ALERT_2_C Short  C(sp3)-C(sp3) Bond  C76      - C97      .       1.35 Ang.
```

Author Response: This alert is caused by severe disorder of the surface ligands.

```
PLAT411_ALERT_2_C Short Inter H...H Contact  H21      ..H83      .       2.03 Ang.
                                            1/2-x,3/2-y,1-z  =      7_566 Check
```

Author Response: This alert is caused by severe disorder of the surface ligands.

Alert level G

```
FORMU01_ALERT_1_G  There is a discrepancy between the atom counts in the
            _chemical_formula_sum and _chemical_formula_moiety. This is
            usually due to the moiety formula being in the wrong format.
            Atom count from _chemical_formula_sum:   C229 H243 Cl3 Cu18 N3 O6 P6 S
            Atom count from _chemical_formula_moiety:C229 H243 Cl3 Cu18 O3 P6 S14
FORMU01_ALERT_2_G  There is a discrepancy between the atom counts in the
            _chemical_formula_sum and the formula from the _atom_site* data.
            Atom count from _chemical_formula_sum:C229 H243 Cl3 Cu18 N3 O6 P6 S14
            Atom count from the _atom_site data:  C220 H216 Cl3 Cu18 P6 S14
CELLZ01_ALERT_1_G Difference between formula and atom_site contents detected.
CELLZ01_ALERT_1_G ALERT: Large difference may be due to a
            symmetry error - see SYMMG tests
           From the CIF: _cell_formula_units_Z    4
           From the CIF: _chemical_formula_sum  C229 H243 Cl3 Cu18 N3 O6 P6 S14
           TEST: Compare cell contents of formula and atom_site data

           atom    Z*formula  cif sites diff
           C        916.00    880.00   36.00
           H        972.00    864.00  108.00
           Cl        12.00     12.00    0.00
           Cu        72.00     72.00    0.00
           N         12.00      0.00   12.00
           O         24.00      0.00   24.00
           P         24.00     24.00    0.00
           S         56.00     56.00    0.00
PLAT002_ALERT_2_G Number of Distance or Angle Restraints on AtSite          2 Note
PLAT003_ALERT_2_G Number of Uiso or Uij Restrained non-H Atoms ...         62 Report
PLAT012_ALERT_1_G No       _shelx_res_checksum Found in CIF ......     Please Check
PLAT041_ALERT_1_G Calc. and Reported SumFormula    Strings  Differ     Please Check
             Calc: C220 H216 Cl3 Cu18 P6 S14
             Rep.: C229 H243 Cl3 Cu18 N3 O6 P6 S14
PLAT042_ALERT_1_G Calc. and Reported MoietyFormula Strings  Differ     Please Check
             Calc: C220 H216 Cl3 Cu18 P6 S14
             Rep.: C220 H216 Cl3 Cu18 P6 S14, 3[C2H3N.CH4O.H2O]
PLAT051_ALERT_1_G Mu(calc) and Mu(CIF) Ratio Differs from 1.0 by .       1.37 %
PLAT072_ALERT_2_G SHELXL First  Parameter in WGHT  Unusually Large       0.20 Report
PLAT172_ALERT_4_G The CIF-Embedded .res File Contains DFIX Records          1 Report
PLAT177_ALERT_4_G The CIF-Embedded .res File Contains DELU Records         12 Report
PLAT178_ALERT_4_G The CIF-Embedded .res File Contains SIMU Records         16 Report
PLAT188_ALERT_3_G A Non-default SIMU Restraint Value has been used     0.0100 Report
PLAT188_ALERT_3_G A Non-default SIMU Restraint Value has been used     0.0100 Report
PLAT188_ALERT_3_G A Non-default SIMU Restraint Value has been used     0.0100 Report
PLAT188_ALERT_3_G A Non-default SIMU Restraint Value has been used     0.0100 Report
PLAT188_ALERT_3_G A Non-default SIMU Restraint Value has been used     0.0100 Report
PLAT188_ALERT_3_G A Non-default SIMU Restraint Value has been used     0.0100 Report
PLAT188_ALERT_3_G A Non-default SIMU Restraint Value has been used     0.0100 Report
PLAT188_ALERT_3_G A Non-default SIMU Restraint Value has been used     0.0100 Report
PLAT188_ALERT_3_G A Non-default SIMU Restraint Value has been used     0.0100 Report
PLAT188_ALERT_3_G A Non-default SIMU Restraint Value has been used     0.0100 Report
PLAT188_ALERT_3_G A Non-default SIMU Restraint Value has been used     0.0100 Report
PLAT188_ALERT_3_G A Non-default SIMU Restraint Value has been used     0.0100 Report
PLAT188_ALERT_3_G A Non-default SIMU Restraint Value has been used     0.0100 Report
PLAT188_ALERT_3_G A Non-default SIMU Restraint Value has been used     0.0100 Report
PLAT188_ALERT_3_G A Non-default SIMU Restraint Value has been used     0.0100 Report
```

```
PLAT188_ALERT_3_G A Non-default SIMU Restraint Value has been used     0.0100 Report
PLAT232_ALERT_2_G Hirshfeld Test Diff (M-X)  Cu2      --S3       .        5.7 s.u.
PLAT232_ALERT_2_G Hirshfeld Test Diff (M-X)  Cu2      --S5       .        5.5 s.u.
PLAT232_ALERT_2_G Hirshfeld Test Diff (M-X)  Cu9      --S7_a     .        6.0 s.u.
PLAT232_ALERT_2_G Hirshfeld Test Diff (M-X)  Cu10     --Cl2      .       14.8 s.u.
PLAT432_ALERT_2_G Short Inter X...Y Contact  C11      ..C89      .       2.47 Ang.
                                            3/2-x,3/2-y,1-z  =      7_666 Check
PLAT432_ALERT_2_G Short Inter X...Y Contact  C89      ..C89      .       2.35 Ang.
                                            3/2-x,3/2-y,1-z  =      7_666 Check
PLAT606_ALERT_4_G Solvent Accessible VOID(S) in Structure .......          ! Info
PLAT794_ALERT_5_G Tentative Bond Valency for Cu1       (I)       .       1.07 Info
PLAT794_ALERT_5_G Tentative Bond Valency for Cu2       (I)       .       1.18 Info
PLAT794_ALERT_5_G Tentative Bond Valency for Cu3       (I)       .       0.75 Info
PLAT794_ALERT_5_G Tentative Bond Valency for Cu4       (I)       .       1.10 Info
PLAT794_ALERT_5_G Tentative Bond Valency for Cu5       (I)       .       1.16 Info
PLAT794_ALERT_5_G Tentative Bond Valency for Cu6       (I)       .       0.76 Info
PLAT794_ALERT_5_G Tentative Bond Valency for Cu7       (I)       .       1.10 Info
PLAT794_ALERT_5_G Tentative Bond Valency for Cu8       (I)       .       1.18 Info
PLAT794_ALERT_5_G Tentative Bond Valency for Cu9       (II)      .       1.82 Info
PLAT794_ALERT_5_G Tentative Bond Valency for Cu10      (I)       .       1.17 Info
PLAT860_ALERT_3_G Number of Least-Squares Restraints ............        464 Note
PLAT868_ALERT_4_G ALERTS Due to the Use of _smtbx_masks Suppressed         ! Info
PLAT933_ALERT_2_G Number of HKL-OMIT Records in Embedded .res File       106 Note
              -13  1  5, -10  2  6,  -9  1  6,  -8  4  1,  -7  5  6,  -7  5  9,
               -6  0  4,  -6  2  1,  -6  2  2,  -6  2  3,  -6  2  4,  -5  1  1,
               -5  1  2,  -5  3  1,  -5  3  2,  -5  3 11,  -4  0  2,  -4  4  1,
               -3  1  3,  -3  1  7,  -3  3  2,  -3  3  4,  -3  3  5,  -2  0  4,
               -2  0  8,  -2  0 12,  -2  2  1,  -2  2  4,  -2  2  7,  -2  4  3,
               -2  4  4,  -2  4  6,  -2  4 10,  -2  6  1,  -2  6  2,  -2  6  4,
               -2  6  8,  -2  8  1,  -2 10  6,  -1  1  1,  -1  3  5,  -1  3  7,
               -1  3  9,  -1  5  4,  -1  5  5,  -1  7  2,   0  0  2,   0  0  6,
                0  2  2,   0  2  3,
```

---

 1 **ALERT level A** = Most likely a serious problem - resolve or explain
12 **ALERT level B** = A potentially serious problem, consider carefully
64 **ALERT level C** = Check. Ensure it is not caused by an omission or oversight
50 **ALERT level G** = General information/check it is not something unexpected

 7 ALERT type 1 CIF construction/syntax error, inconsistent or missing data
67 ALERT type 2 Indicator that the structure model may be wrong or deficient
20 ALERT type 3 Indicator that the structure quality may be low
23 ALERT type 4 Improvement, methodology, query or suggestion
10 ALERT type 5 Informative message, check

It is advisable to attempt to resolve as many as possible of the alerts in all categories. Often the minor alerts point to easily fixed oversights, errors and omissions in your CIF or refinement strategy, so attention to these fine details can be worthwhile. In order to resolve some of the more serious problems it may be necessary to carry out additional measurements or structure refinements. However, the purpose of your study may justify the reported deviations and the more serious of these should normally be commented upon in the discussion or experimental section of a paper or in the "special_details" fields of the CIF. checkCIF was carefully designed to identify outliers and unusual parameters, but every test has its limitations and alerts that are not important in a particular case may appear. Conversely, the absence of alerts does not guarantee there are no aspects of the results needing attention. It is up to the individual to critically assess their own results and, if necessary, seek expert advice.

Publication of your CIF in IUCr journals

A basic structural check has been run on your CIF. These basic checks will be run on all CIFs submitted for publication in IUCr journals (*Acta Crystallographica*, *Journal of Applied Crystallography*, *Journal of Synchrotron Radiation*); however, if you intend to submit to *Acta Crystallographica Section C* or *E* or *IUCrData*, you should make sure that full publication checks are run on the final version of your CIF prior to submission.

Publication of your CIF in other journals

Please refer to the *Notes for Authors* of the relevant journal for any special instructions relating to CIF submission.

PLATON version of 06/01/2024; check.def file version of 05/01/2024

Datablock cu2202 - ellipsoid plot

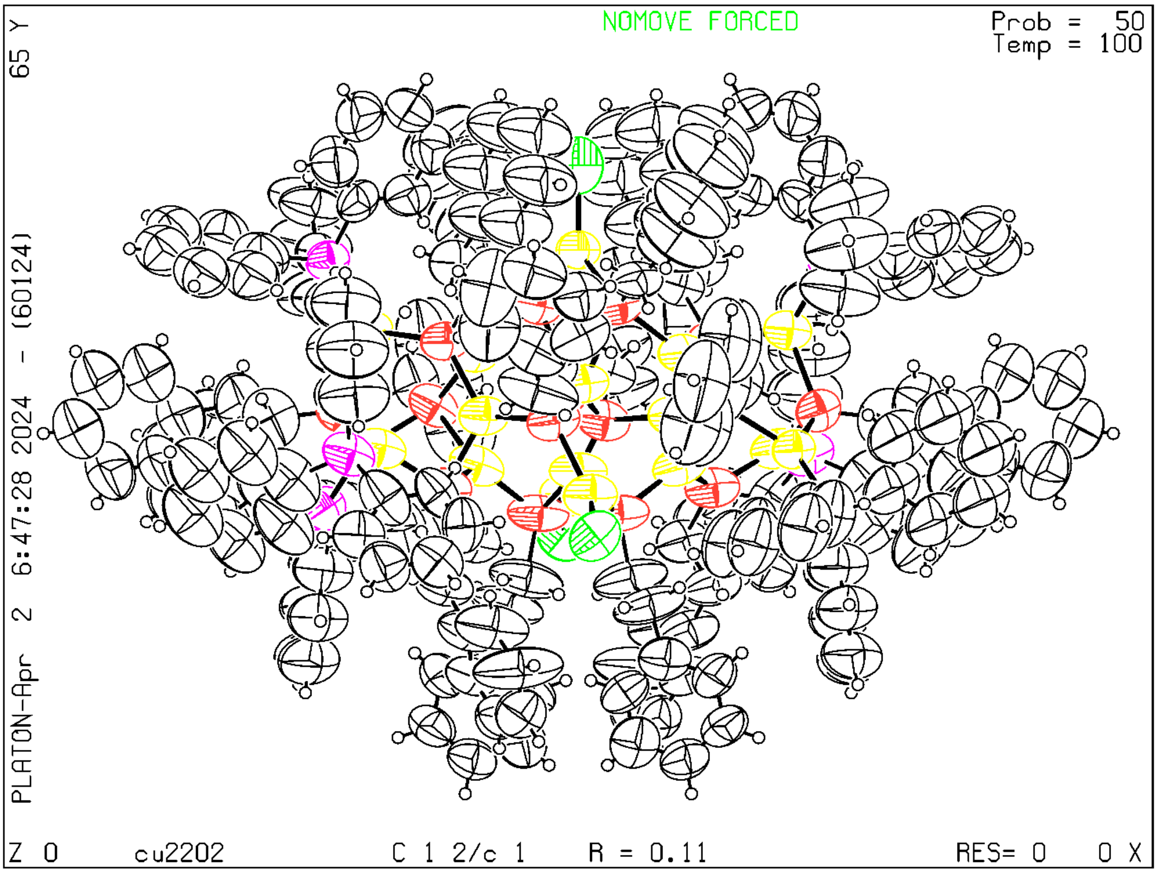

Supplement: Supplementary file 1 — tz4c02148_si_001.pdf [file tz4c02148_si_001.pdf]
