# Supplementary material for: Polymorphism of [Cu15(PhCH2CH2S)13(PPh3)6][BF4]2 and Double-Helical Assembly of [Cu18H(PhCH2CH2S)14(PPh3)6Cl3]: Origin of Two Chiral Nanoclusters with Triple-Helical Core from Intermediates
Source: ACS Mater Lett. 2025 Jan 2;7(2):442–9. doi: 10.1021/acsmaterialslett.4c02148 (PMC11795624; doi:10.1021/acsmaterialslett.4c02148)

checkCIF/PLATON report

You have not supplied any structure factors. As a result the full set of tests cannot be run.

THIS REPORT IS FOR GUIDANCE ONLY. IF USED AS PART OF A REVIEW PROCEDURE FOR PUBLICATION, IT SHOULD NOT REPLACE THE EXPERTISE OF AN EXPERIENCED CRYSTALLOGRAPHIC REFEREE.

No syntax errors found.   CIF dictionary   Interpreting this report

Datablock: cu15

```
Bond precision:   C-C = 0.0419 A          Wavelength=1.54184

Cell:           a=18.8140(3)        b=32.3256(5)        c=38.2569(9)
                alpha=112.773(2)    beta=102.902(2)     gamma=90.426(1)
Temperature:    100 K
```

	Calculated	Reported
Volume	20797.1(8)	20797.1(7)
Space group	P -1	P -1
Hall group	-P 1	-P 1
Moiety formula	C212 H207 Cu15 P6 S13 [+ solvent]	C212 H207 Cu15 P6 S13, [+ solventS]
Sum formula	C212 H207 Cu15 P6 S13 [+ solvent]	C218.43 H222.25 B0.56 Cl1.88 Cu15 F2.25 N8.62 O0.06 P6 S13
Mr	4310.59	4640.24
Dx,g cm-3	1.377	1.482
Z	4	4
Mu (mm-1)	3.638	3.926
F000	8848.0	9527.0
F000'	8768.68	
h,k,lmax	22,38,45	22,38,45
Nref	74753	73536
Tmin,Tmax	0.450,0.855	0.717,1.000
Tmin'	0.343	

```
Correction method= # Reported T Limits: Tmin=0.717 Tmax=1.000
AbsCorr = GAUSSIAN

Data completeness= 0.984          Theta(max)= 67.341
```

```
R(reflections)= 0.2189( 51696)                    wR2(reflections)=
                                                  0.5805( 73536)
S = 2.415                  Npar= 4274
```

The following ALERTS were generated. Each ALERT has the format
test-name_ALERT_alert-type_alert-level.
Click on the hyperlinks for more details of the test.

Alert level A

```
PLAT082_ALERT_2_A High R1 Value ................................       0.22 Report
```

Author Response: This alert is caused by week diffraction of the crystal and severe disorder of the surface ligands as well,even though multiple attempts were made to grow better diffracting crystals.

```
PLAT084_ALERT_3_A High wR2 Value (i.e. > 0.25) ..................       0.58 Report
```

Author Response: This alert is caused by week diffraction of the crystal and severe disorder of the surface ligands as well,even though multiple attempts were made to grow better diffracting crystals.

```
PLAT411_ALERT_2_A Short Inter H...H Contact  H315     ..H635     .       1.43 Ang.
                                               1-x,2-y,-z  =      2_675 Check
```

Author Response: This alert is caused by week diffraction of the crystal and severe disorder of the phneyl ring.

Alert level B

```
PLAT341_ALERT_3_B Low Bond Precision on  C-C Bonds ..............    0.04189 Ang.
```

Author Response: This alert is caused by week diffraction of the crystal and severe disorder of the surface ligands as well,even though multiple attempts were made to grow better diffracting crystals.

```
PLAT369_ALERT_2_B Long   C(sp2)-C(sp2) Bond  C324     - C576     .       1.63 Ang.
```

Author Response: This alert is caused by week diffraction of the crystal and severe disorder of the surface ligands as well,even though multiple attempts were made to grow better diffracting crystals.

Alert level C

```
DIFMX02_ALERT_1_C  The maximum difference density is > 0.1*ZMAX*0.75
            The relevant atom site should be identified.
GOODF01_ALERT_2_C  The least squares goodness of fit parameter lies
            outside the range 0.80 <> 2.00
            Goodness of fit given =      2.415
PLAT087_ALERT_2_C Unsatisfactory S value (Too High) .............       2.41 Check
PLAT097_ALERT_2_C Large Reported Max.  (Positive) Residual Density       2.68 eA-3
PLAT213_ALERT_2_C Atom C352            has ADP max/min Ratio .....        3.3 oblate
PLAT213_ALERT_2_C Atom C464            has ADP max/min Ratio .....        3.7 oblate
PLAT213_ALERT_2_C Atom C546            has ADP max/min Ratio .....        3.1 oblate
PLAT213_ALERT_2_C Atom C225            has ADP max/min Ratio .....        3.1 prolat
PLAT213_ALERT_2_C Atom C272            has ADP max/min Ratio .....        3.3 prolat
PLAT213_ALERT_2_C Atom C396            has ADP max/min Ratio .....        3.3 prolat
PLAT213_ALERT_2_C Atom C471            has ADP max/min Ratio .....        3.4 prolat
PLAT213_ALERT_2_C Atom C621            has ADP max/min Ratio .....        3.3 prolat
PLAT220_ALERT_2_C NonSolvent   Resd 2  C   Ueq(max)/Ueq(min) Range        4.6 Ratio
PLAT222_ALERT_3_C NonSolvent Resd 2  H   Uiso(max)/Uiso(min) Range        4.6 Ratio
PLAT241_ALERT_2_C High   'MainMol' Ueq as Compared to Neighbors of       C159 Check
PLAT241_ALERT_2_C High   'MainMol' Ueq as Compared to Neighbors of       C528 Check
PLAT241_ALERT_2_C High   'MainMol' Ueq as Compared to Neighbors of       C396 Check
PLAT241_ALERT_2_C High   'MainMol' Ueq as Compared to Neighbors of       C475 Check
PLAT241_ALERT_2_C High   'MainMol' Ueq as Compared to Neighbors of       C542 Check
PLAT242_ALERT_2_C Low    'MainMol' Ueq as Compared to Neighbors of         P4 Check
PLAT242_ALERT_2_C Low    'MainMol' Ueq as Compared to Neighbors of        P18 Check
PLAT242_ALERT_2_C Low    'MainMol' Ueq as Compared to Neighbors of        S25 Check
PLAT242_ALERT_2_C Low    'MainMol' Ueq as Compared to Neighbors of        S37 Check
PLAT242_ALERT_2_C Low    'MainMol' Ueq as Compared to Neighbors of         P3 Check
PLAT242_ALERT_2_C Low    'MainMol' Ueq as Compared to Neighbors of         P5 Check
PLAT242_ALERT_2_C Low    'MainMol' Ueq as Compared to Neighbors of        P10 Check
PLAT250_ALERT_2_C Large U3/U1 Ratio for <U(i,j)> Tensor(Resd    2)        2.3 Note
PLAT330_ALERT_2_C Large Aver Phenyl C-C Dist C113     --C490     .       1.42 Ang.
PLAT331_ALERT_2_C Small Aver Phenyl C-C Dist C50      --C514     .       1.37 Ang.
PLAT332_ALERT_2_C Large Phenyl C-C Range     C4        -C354     .       0.21 Ang.
PLAT332_ALERT_2_C Large Phenyl C-C Range     C5        -C498     .       0.18 Ang.
PLAT332_ALERT_2_C Large Phenyl C-C Range     C20       -C552     .       0.23 Ang.
PLAT332_ALERT_2_C Large Phenyl C-C Range     C36       -C342     .       0.23 Ang.
PLAT332_ALERT_2_C Large Phenyl C-C Range     C42       -C504     .       0.23 Ang.
PLAT332_ALERT_2_C Large Phenyl C-C Range     C43       -C524     .       0.16 Ang.
PLAT332_ALERT_2_C Large Phenyl C-C Range     C70       -C463     .       0.24 Ang.
PLAT332_ALERT_2_C Large Phenyl C-C Range     C107      -C425     .       0.16 Ang.
PLAT332_ALERT_2_C Large Phenyl C-C Range     C190      -C367     .       0.20 Ang.
PLAT360_ALERT_2_C Short  C(sp3)-C(sp3) Bond  C180     - C298     .       1.41 Ang.
PLAT360_ALERT_2_C Short  C(sp3)-C(sp3) Bond  C471     - C513     .       1.38 Ang.
PLAT361_ALERT_2_C Long   C(sp3)-C(sp3) Bond  C396     - C611     .       1.67 Ang.
PLAT362_ALERT_2_C Short  C(sp3)-C(sp2) Bond  C87      - C201     .       1.41 Ang.
PLAT363_ALERT_2_C Long   C(sp3)-C(sp2) Bond  C611     - C631     .       1.68 Ang.
PLAT368_ALERT_2_C Short  C(sp2)-C(sp2) Bond  C264     - C401     .       1.18 Ang.
PLAT368_ALERT_2_C Short  C(sp2)-C(sp2) Bond  C344     - C422     .       1.22 Ang.
PLAT368_ALERT_2_C Short  C(sp2)-C(sp2) Bond  C324     - C433     .       1.23 Ang.
PLAT369_ALERT_2_C Long   C(sp2)-C(sp2) Bond  C36      - C342     .       1.53 Ang.
```

Author Response: This alert is caused by week diffraction of the crystal and severe disorder of the surface ligands as well,even though multiple attempts were made to grow better diffracting crystals.

```
PLAT369_ALERT_2_C Long   C(sp2)-C(sp2) Bond  C248     - C504     .       1.53 Ang.
```

Author Response: This alert is caused by week diffraction of the crystal and severe disorder of the surface ligands as well,even though multiple attempts were made to grow better diffracting crystals.

```
PLAT369_ALERT_2_C Long   C(sp2)-C(sp2) Bond  C363     - C434     .       1.54 Ang.
```

Author Response: This alert is caused by week diffraction of the crystal and severe disorder of the surface ligands as well,even though multiple attempts were made to grow better diffracting crystals.

```
PLAT369_ALERT_2_C Long   C(sp2)-C(sp2) Bond  C371     - C614     .       1.53 Ang.
```

Author Response: This alert is caused by week diffraction of the crystal and severe disorder of the surface ligands as well,even though multiple attempts were made to grow better diffracting crystals.

```
PLAT369_ALERT_2_C Long   C(sp2)-C(sp2) Bond  C289     - C603     .       1.54 Ang.
```

Author Response: This alert is caused by week diffraction of the crystal and severe disorder of the surface ligands as well,even though multiple attempts were made to grow better diffracting crystals.

Alert level G

```
FORMU01_ALERT_1_G  There is a discrepancy between the atom counts in the
            _chemical_formula_sum and _chemical_formula_moiety. This is
            usually due to the moiety formula being in the wrong format.
            Atom count from _chemical_formula_sum:   C218.4299 H222.25 B0.56 Cl1.8
            Atom count from _chemical_formula_moiety:C212 H207 Cu15 P6 S13
FORMU01_ALERT_2_G  There is a discrepancy between the atom counts in the
            _chemical_formula_sum and the formula from the _atom_site* data.
            Atom count from _chemical_formula_sum:C218.4299 H222.25 B0.56 Cl1.88 C
            Atom count from the _atom_site data:  C212 H207 Cu15 P6 S13
CELLZ01_ALERT_1_G Difference between formula and atom_site contents detected.
CELLZ01_ALERT_1_G ALERT: Large difference may be due to a
            symmetry error - see SYMMG tests
           From the CIF: _cell_formula_units_Z    4
           From the CIF: _chemical_formula_sum  C218.43 H222.25 B0.56 Cl1.88 Cu15
           TEST: Compare cell contents of formula and atom_site data

           atom    Z*formula  cif sites diff
```

```
          C        873.72    848.00   25.72
          H        889.00    828.00   61.00
          B          2.24      0.00    2.24
          Cl         7.52      0.00    7.52
          Cu        60.00     60.00    0.00
          F          9.00      0.00    9.00
          N         34.48      0.00   34.48
          O          0.24      0.00    0.24
          P         24.00     24.00    0.00
          S         52.00     52.00    0.00
PLAT002_ALERT_2_G Number of Distance or Angle Restraints on AtSite          6 Note
PLAT003_ALERT_2_G Number of Uiso or Uij Restrained non-H Atoms ...        424 Report
PLAT012_ALERT_1_G No       _shelx_res_checksum Found in CIF ......     Please Check
PLAT041_ALERT_1_G Calc. and Reported SumFormula    Strings  Differ     Please Check
             Calc: C212 H207 Cu15 P6 S13
             Rep.: C218.43 H222.25 B0.56 Cl1.88 Cu15 F2.25 N8.62
                   O0.06 P6 S13
PLAT042_ALERT_1_G Calc. and Reported MoietyFormula Strings  Differ     Please Check
             Calc: C212 H207 Cu15 P6 S13
             Rep.: C212 H207 Cu15 P6 S13,
PLAT051_ALERT_1_G Mu(calc) and Mu(CIF) Ratio Differs from 1.0 by .       7.34 %
PLAT072_ALERT_2_G SHELXL First  Parameter in WGHT  Unusually Large       0.20 Report
PLAT172_ALERT_4_G The CIF-Embedded .res File Contains DFIX Records          3 Report
PLAT173_ALERT_4_G The CIF-Embedded .res File Contains DANG Records          3 Report
PLAT174_ALERT_4_G The CIF-Embedded .res File Contains FLAT Records          1 Report
PLAT176_ALERT_4_G The CIF-Embedded .res File Contains SADI Records          2 Report
PLAT178_ALERT_4_G The CIF-Embedded .res File Contains SIMU Records          4 Report
PLAT186_ALERT_4_G The CIF-Embedded .res File Contains ISOR Records          4 Report
PLAT187_ALERT_4_G The CIF-Embedded .res File Contains RIGU Records          2 Report
PLAT188_ALERT_3_G A Non-default SIMU Restraint Value has been used     0.0100 Report
PLAT188_ALERT_3_G A Non-default SIMU Restraint Value has been used     0.0100 Report
PLAT191_ALERT_3_G A Non-default SADI Restraint Value has been used     0.0400 Report
PLAT343_ALERT_2_G Unusual sp3      Angle Range in Main Residue for       C405 Check
PLAT432_ALERT_2_G Short Inter X...Y Contact  C315     ..C635     .       2.76 Ang.
                                                1-x,2-y,-z  =     2_675 Check
PLAT432_ALERT_2_G Short Inter X...Y Contact  C315     ..C315     .       2.98 Ang.
                                                1-x,2-y,-z  =     2_675 Check
PLAT432_ALERT_2_G Short Inter X...Y Contact  C635     ..C635     .       3.18 Ang.
                                                1-x,2-y,-z  =     2_675 Check
PLAT606_ALERT_4_G Solvent Accessible VOID(S) in Structure ........          ! Info
PLAT794_ALERT_5_G Tentative Bond Valency for Cu1       (I)       .       1.12 Info
PLAT794_ALERT_5_G Tentative Bond Valency for Cu4       (I)       .       0.71 Info
PLAT794_ALERT_5_G Tentative Bond Valency for Cu6       (I)       .       0.71 Info
PLAT794_ALERT_5_G Tentative Bond Valency for Cu8       (I)       .       1.11 Info
PLAT794_ALERT_5_G Tentative Bond Valency for Cu14      (I)       .       0.72 Info
PLAT794_ALERT_5_G Tentative Bond Valency for Cu15      (I)       .       1.09 Info
PLAT794_ALERT_5_G Tentative Bond Valency for Cu16      (I)       .       1.08 Info
PLAT794_ALERT_5_G Tentative Bond Valency for Cu18      (I)       .       1.06 Info
PLAT794_ALERT_5_G Tentative Bond Valency for Cu20      (I)       .       1.06 Info
PLAT794_ALERT_5_G Tentative Bond Valency for Cu22      (I)       .       1.05 Info
PLAT794_ALERT_5_G Tentative Bond Valency for Cu26      (I)       .       1.06 Info
PLAT794_ALERT_5_G Tentative Bond Valency for Cu28      (I)       .       1.00 Info
PLAT794_ALERT_5_G Tentative Bond Valency for Cu30      (I)       .       1.07 Info
PLAT794_ALERT_5_G Tentative Bond Valency for Cu31      (I)       .       0.71 Info
PLAT794_ALERT_5_G Tentative Bond Valency for Cu33      (I)       .       1.07 Info
PLAT794_ALERT_5_G Tentative Bond Valency for Cu34      (I)       .       0.72 Info
PLAT794_ALERT_5_G Tentative Bond Valency for Cu35      (I)       .       0.70 Info
```

```
PLAT794_ALERT_5_G Tentative Bond Valency for Cu36      (I)       .       1.02 Info
PLAT794_ALERT_5_G Tentative Bond Valency for Cu38      (I)       .       1.05 Info
PLAT794_ALERT_5_G Tentative Bond Valency for Cu40      (I)       .       1.08 Info
PLAT794_ALERT_5_G Tentative Bond Valency for Cu41      (I)       .       0.99 Info
PLAT794_ALERT_5_G Tentative Bond Valency for Cu42      (I)       .       1.08 Info
PLAT794_ALERT_5_G Tentative Bond Valency for Cu43      (I)       .       1.03 Info
PLAT794_ALERT_5_G Tentative Bond Valency for Cu44      (I)       .       1.07 Info
PLAT794_ALERT_5_G Tentative Bond Valency for Cu45      (I)       .       1.05 Info
PLAT860_ALERT_3_G Number of Least-Squares Restraints ............       2648 Note
PLAT868_ALERT_4_G ALERTS Due to the Use of _smtbx_masks Suppressed          ! Info
PLAT870_ALERT_4_G ALERTS Related to Twinning Effects Suppressed ..          ! Info
PLAT933_ALERT_2_G Number of HKL-OMIT Records in Embedded .res File        105 Note
              -10 -8  3,  -9  0 13,  -9  0 15,  -9  3  1,  -8 -2  1,  -8  0  2,
               -7-14 12,  -7 -6  4,  -7 -5  2,  -6-13 10,  -6-13 12,  -6-13 14,
               -6-10 15,  -6 -9 11,  -6  3  6,  -5-14  9,  -5-14 11,  -5-12 10,
               -5-11 10,  -5-10  1,  -5 -7 10,  -5 -5 10,  -5  2  5,  -5  3  1,
               -5  4  4,  -5  6  5,  -5  7  2,  -4-13  9,  -4 -9  2,  -4 -3  5,
               -4 -1  3,  -4  0  2,  -4  0  4,  -4  1  2,  -4  3  4,  -4 13  0,
               -3-13  9,  -3-12 15,  -3-11  7,  -3 -5  6,  -3 -5 17,  -3 14  0,
               -2-12  8,  -2 -9  5,  -2 -6  6,  -2  0  1,  -2  2  1,  -2  3  4,
               -2  5  4,  -1-15  7,
```

---

3 **ALERT level A** = Most likely a serious problem - resolve or explain
2 **ALERT level B** = A potentially serious problem, consider carefully
51 **ALERT level C** = Check. Ensure it is not caused by an omission or oversight
55 **ALERT level G** = General information/check it is not something unexpected

8 ALERT type 1 CIF construction/syntax error, inconsistent or missing data
61 ALERT type 2 Indicator that the structure model may be wrong or deficient
7 ALERT type 3 Indicator that the structure quality may be low
10 ALERT type 4 Improvement, methodology, query or suggestion
25 ALERT type 5 Informative message, check

It is advisable to attempt to resolve as many as possible of the alerts in all categories. Often the minor alerts point to easily fixed oversights, errors and omissions in your CIF or refinement strategy, so attention to these fine details can be worthwhile. In order to resolve some of the more serious problems it may be necessary to carry out additional measurements or structure refinements. However, the purpose of your study may justify the reported deviations and the more serious of these should normally be commented upon in the discussion or experimental section of a paper or in the "special_details" fields of the CIF. checkCIF was carefully designed to identify outliers and unusual parameters, but every test has its limitations and alerts that are not important in a particular case may appear. Conversely, the absence of alerts does not guarantee there are no aspects of the results needing attention. It is up to the individual to critically assess their own results and, if necessary, seek expert advice.

Publication of your CIF in IUCr journals

A basic structural check has been run on your CIF. These basic checks will be run on all CIFs submitted for publication in IUCr journals (*Acta Crystallographica*, *Journal of Applied Crystallography*, *Journal of Synchrotron Radiation*); however, if you intend to submit to *Acta Crystallographica Section C* or *E* or *IUCrData*, you should make sure that full publication checks are run on the final version of your CIF prior to submission.

Publication of your CIF in other journals

Please refer to the *Notes for Authors* of the relevant journal for any special instructions relating to CIF submission.

PLATON version of 06/01/2024; check.def file version of 05/01/2024

Datablock cu15 - ellipsoid plot

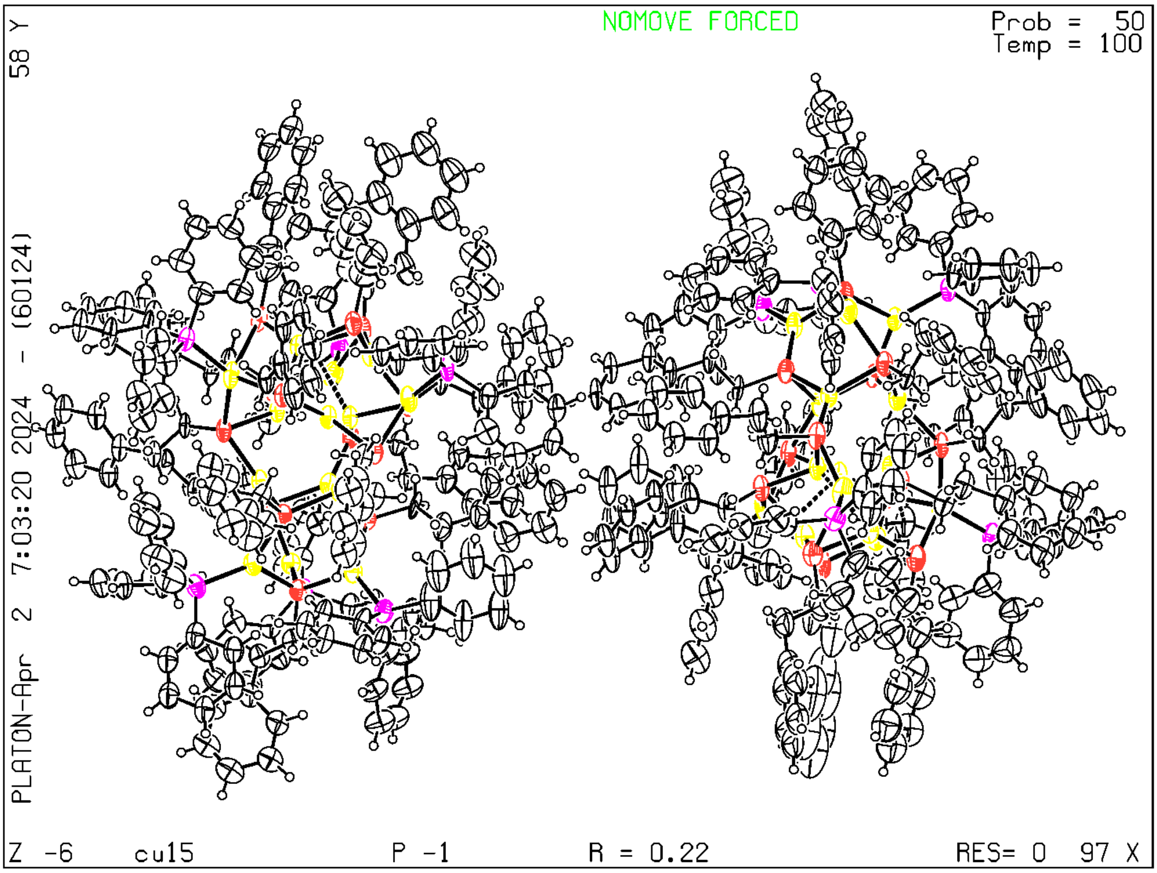

Supplement: Supplementary file 2 — tz4c02148_si_002.pdf [file tz4c02148_si_002.pdf]
