# Supplementary material for: Polymorphism of [Cu15(PhCH2CH2S)13(PPh3)6][BF4]2 and Double-Helical Assembly of [Cu18H(PhCH2CH2S)14(PPh3)6Cl3]: Origin of Two Chiral Nanoclusters with Triple-Helical Core from Intermediates
Source: ACS Mater Lett. 2025 Jan 2;7(2):442–9. doi: 10.1021/acsmaterialslett.4c02148 (PMC11795624; doi:10.1021/acsmaterialslett.4c02148)
Supplement: Supplementary file 6 — tz4c02148_si_006.pdf [file tz4c02148_si_006.pdf]

Supporting Information

Polymorphism of $[Cu_{15}(PhCH_2CH_2S)_{13}(PPh_3)_6][BF_4]_2$ and Double-Helical Assembly of $[Cu_{18}H(PhCH_2CH_2S)_{14}(PPh_3)_6Cl_3]$: Origin of Two Chiral Nanoclusters with Triple-Helical Core from Intermediates

Abhijit Nag†, ‡, Abdul Mannan Butt†, ‡, Moon Young Yang§, Praveen B. Managutti‡, #, Bilal Masood Pirzada †‡, M. Infas H. Mohideen†, ‡, Ahmed L. Abdelhady†, ‡, Mohamed Abu Haija†, ‡, Sharmarke Mohamed†, ‡, #, Boris V. Merinov§, William A. Goddard III§ and Ahsanulhaq Qurashi†, ‡**

† Department of Chemistry, Khalifa University of Science and Technology, Abu Dhabi 127788, UAE

‡ Center for Catalysis and Separations, Khalifa University of Science and Technology, Abu Dhabi 127788, UAE

Chemical Crystallography Laboratory, Khalifa University of Science and Technology, Abu Dhabi, P.O. Box 127788, UAE

§ Materials and Process Simulation Center (MSC). California Institute of Technology, Pasadena CA 91125, USA

*Email: ahsan.qurashi@ku.ac.ae, wag@wag.caltech.edu; Tel: +971 2312 4202

Experimental Section

Materials

Cuprous oxide, tetrafluoroboric acid solution (HBF_4, 48 wt % in water), PETH, PPh_3, sodium borohydride ($NaBH_4$), and high performance liquid chromatography (HPLC) grade solvents (acetonitrile, chloroform, methanol, and hexane) were purchased from Sigma-Aldrich. All chemicals were used directly without further purification.

Synthesis of [Cu(CH_3CN)$_4$]BF_4 complex

Synthesis of [Cu(CH_3CN)$_4$]BF_4 was carried out following a previous publication. [1] 1 g of Cu_2O and 25 mL of CH_3CN was placed in a round bottom flask in the oil bath at 70°C. After 3-5 minutes of stirring, 5 mL of the HBF_4 was added to the solution. The stirring was continued for the next 10-15 min. Later, the hot transparent solution was filtered quickly using a filter paper, and the filtrate was kept in the fridge (0 °C). After 12 h, white crystalline complex, [Cu(CH_3CN)$_4$]BF_4, was collected through filtration followed by two times washing with diethyl ether. Then the complex was transferred to a sample bottle and stored under vacuum. The complex is air sensitive; keeping the copper complex in a closed vial under ambient conditions is enough to use it for over two months.

Synthesis of Cu_{15} and Cu_{18} NC

First, 92 mg of Cu(CH_3CN)$_4BF_4$ and 100 mg of PPh_3 were dissolved in a solvent mixture of acetonitrile (4 mL) and chloroform (1 mL). Then, 20 µL of PETH was added. After 5 min, 100 mg of $NaBH_4$ dissolved in 5 mL of methanol was quickly added to the above mixture, and the solution was vigorously stirred for 30 mins. The precipitate was collected via centrifugation. The precipitate was kept for crystallization with the 200 µL mixture of acetonitrile, chloroform and methanol (4:1:5) in freeze. After two days, formation of Cu_{18} crystals and polymorphic crystals of Cu_{15} were noticed. If the reaction was continued for 5 hr, the formation of only Cu_{15} NC was noticed.

Instrumentation

Electrospray ionization mass spectrometry (ESI-MS)

LC-MS Q Exactive Quadrupole-Orbitrap Mass Spectrometer from the Thermo Fisher Scientific was used for the mass measurement. The NC samples were dissolved in DCM and the solution was injected directly. The instrument is operated in the mass range of m/z 50–6000 for positive mode and negative mode, respectively. The instrument parameters were maintained as follows: Resolution: 70,000, capillary voltage: 3.0 kV, Aux gas: 10 L/min, Auxiliary gas temperature: 200 °C.

X-ray photoelectron spectroscopy (XPS)

XPS measurements were performed in a Kratos Axis Ultra DLD spectrometer equipped with a monochromatic Al K_α X-ray source (hν = 1486.6 eV) operating at 150 W, 0.5 mm circular spot size, a flood gun to counter charging effects, and the S3 analysis chamber, a multi-channel plate and delay line detector under a vacuum of ~10^{-9} mbar. All spectra were recorded using an aperture slot of 300 µm x 700 µm. The Survey spectra and high-resolution spectra were acquired by keeping the pass energy of

160 eV and a step size of 1 eV. To avoid differential charging both compounds were arranged in floating mode. Charge neutralization was performed for both samples. Binding energies of C 1s were used as reference and set at 284 eV. The samples were dried properly before creating the vacuum.

Single-crystals X-ray diffraction (SCXRD)

Single crystals of Cu_{15} were crystallized from methanol, acetonitrile and chloroform using slow solvent evaporation method. A suitable crystal was selected and mounted on a Rigaku Oxford Diffraction XtaLAB Synergy-S diffractometer equipped with a HyPix-6000HE Hybrid Photon Counting (HPC) detector and Cu microfocus sealed X-ray tube, as well as a low-temperature Oxford Cryosystems Cobra low temperature device. The crystal was kept at 99.8(4) K during data collection. The data collection strategy was calculated within CrysAlisP (Rigaku OD, 2024; Table S1) to ensure desired data redundancy and percent completeness. Using Olex2 [2], the structure was solved with the SHELXT [3] structure solution program using Intrinsic Phasing and refined with the SHELXL [4] refinement package using Least Squares minimization. The space group determination was performed by using PLATON [5]. ORTEP diagrams were generated using ORTEP-3 [6]. All non-hydrogen atoms were located in difference-Fourier maps and were then refined anisotropically. All hydrogen atoms were assigned isotropic displacement coefficients U(H) of 1.2U, and their coordinates were allowed to ride on their respective atoms.

The Cu15 was found to be a non merohedral twin with a matrix,

$$\begin{bmatrix} -1 & 0 & 0 \\ 0 & 0.964 & -0.071 \\ 0 & -0.982 & -0.964 \end{bmatrix}$$

and a BASF parameter of 0.0199(8).

Least-squares refinement of the structural model was performed under geometric restraints (SADI) and displacement parameter restraints (RIGU, ISOR, and SIMU) for the surface ligands; of these ligands, five were constrained to idealized hexagons using the AFIX 66 command owing to the highly distorted configurations of the phenyl rings of triphenyl phosphine and eight of the phenyl ethyl thiol (PET). The PET contained atoms C315, C631, C633, C317, C317, C635 is highly disorder and occupancy has been fixed to 1. A large space remains between the nanoclusters after assembly, which is occupied by highly disordered solvent molecules (acetonitrile, methanol, chloroform and BF4). These molecules cannot be directly identified from the difference-Fourier map because of the absence of an assignable model. Therefore, the SQUEEZE program implemented in Olex2 was used to remove the electron densities. The identification of Cu_{15} as a polymorph of the NC reported by Baker et al [7] is therefore based on the NC, which is the same in composition to that reported by Bakr et al but shows distinct crystal packing. The final molecular formula Cu_{15} was supported via ESI-MS, and XPS.

Single crystals of Cu_{18} were crystallized from methanol, acetonitrile and chloroform using slow solvent evaporation method. Suitable crystals were selected and mounted on a Rigaku Oxford Diffraction XtaLAB Synergy-S diffractometer equipped with a HyPix-6000HE Hybrid Photon Counting (HPC)

detector and Cu microfocus sealed X-ray tube, as well as a low-temperature Oxford Cryosystems Cobra low temperature device. The crystal was kept at 99.8(5) K during data collection. The data collection strategy was calculated within CrysAlisP (Rigaku OD, 2024) to ensure desired data redundancy and percent completeness. Using Olex2 [2], the structure was solved with the SHELXT [3] structure solution program using Intrinsic Phasing and refined with the SHELXL [4] refinement package using Least Squares minimization. The space group determination was performed by using PLATON [5]. ORTEP diagrams were generated using ORTEP-3 [6]. All non-hydrogen atoms were located in difference-Fourier maps and were then refined anisotropically. All hydrogen atoms were assigned isotropic displacement coefficients U(H) of 1.2U, and their coordinates were allowed to ride on their respective atoms. Least-squares refinement of the structural model was performed under displacement parameter restraints (SIMU and DELU) for the surface ligands. A large space remains between the nanoclusters after assembly, which is occupied by highly disordered solvent molecules. These molecules cannot be directly identified from the difference-Fourier map because of the absence of an assignable model. Therefore, the SQUEEZE program implemented in Olex2 was used to remove the electron densities. The final molecular formula Cu_{18} was supported via ESI-MS, and XPS.

Computational details

The density functional theory (DFT) calculations were performed using the Gaussian16 program[8] to determine the hydride location on Cu_{18}. Geometry optimization was performed using the B3LYP functional with the D3 correction [9]. We used the LanL2DZ Effective Core Potential for Cu, the 6-31G basis set for P, S, and Cl, and the 3-21G basis set for C and H [10-11].

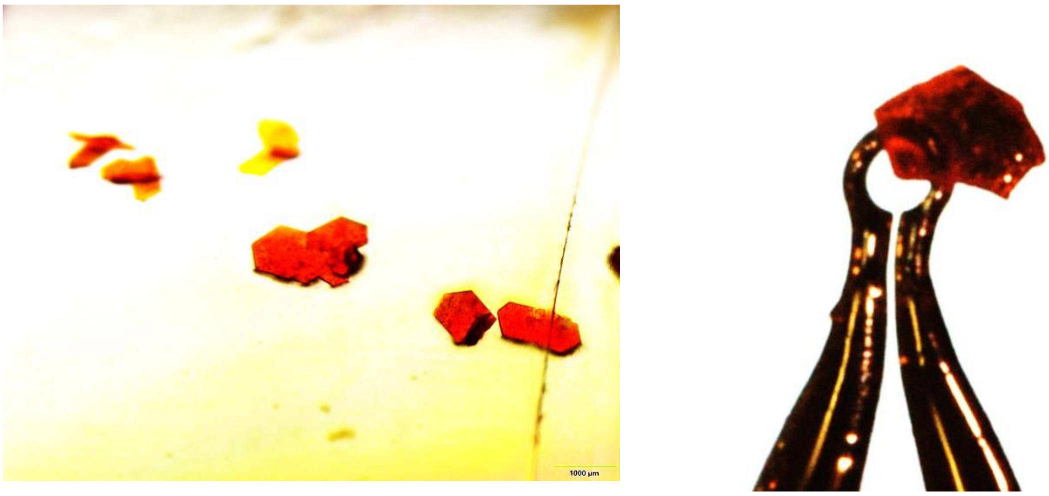

Figure S1. Images of Cu_{15} NC crystals.

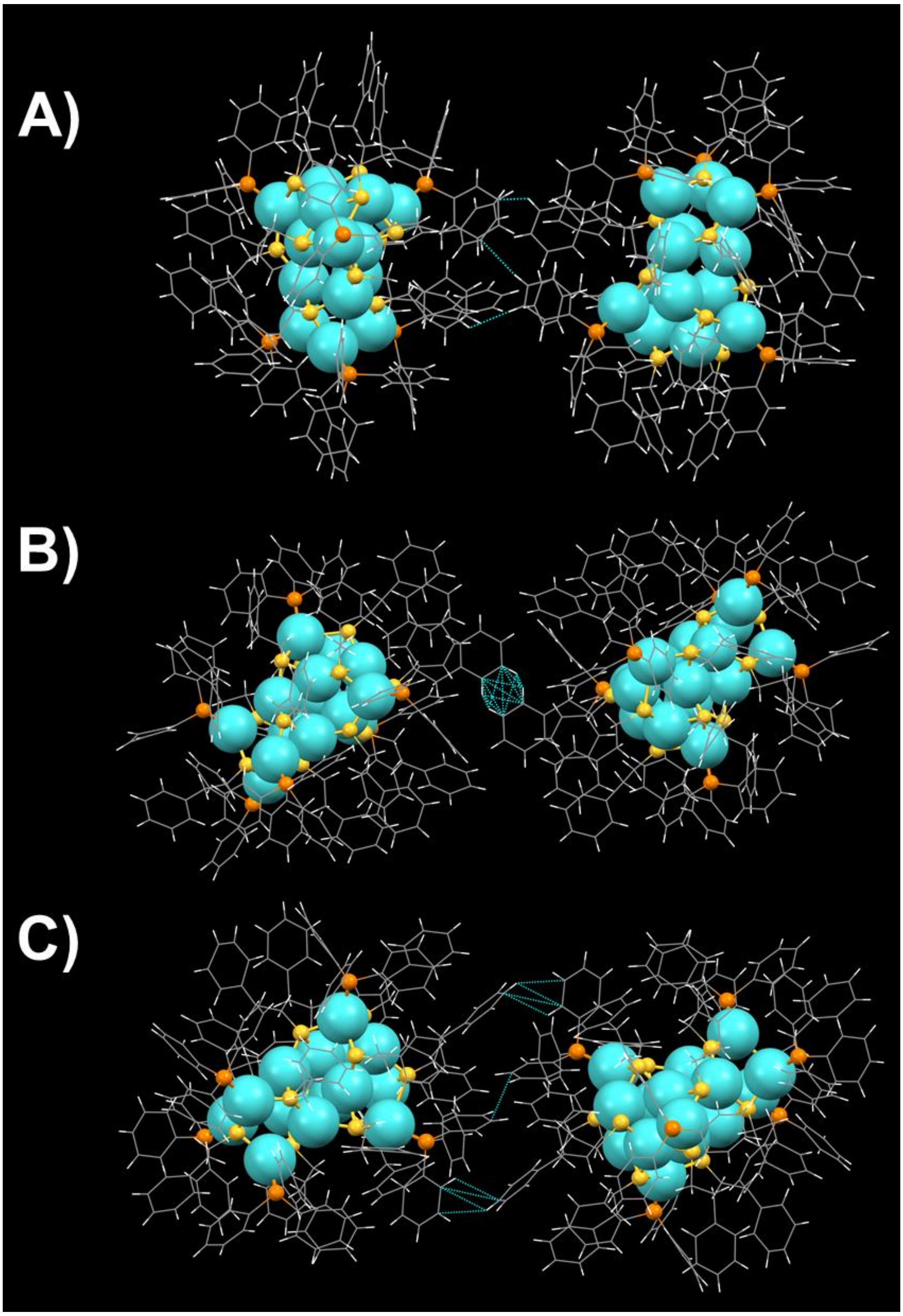

Figure S2. Weak intermolecular C-H···π interactions of Cu_{15} NC.

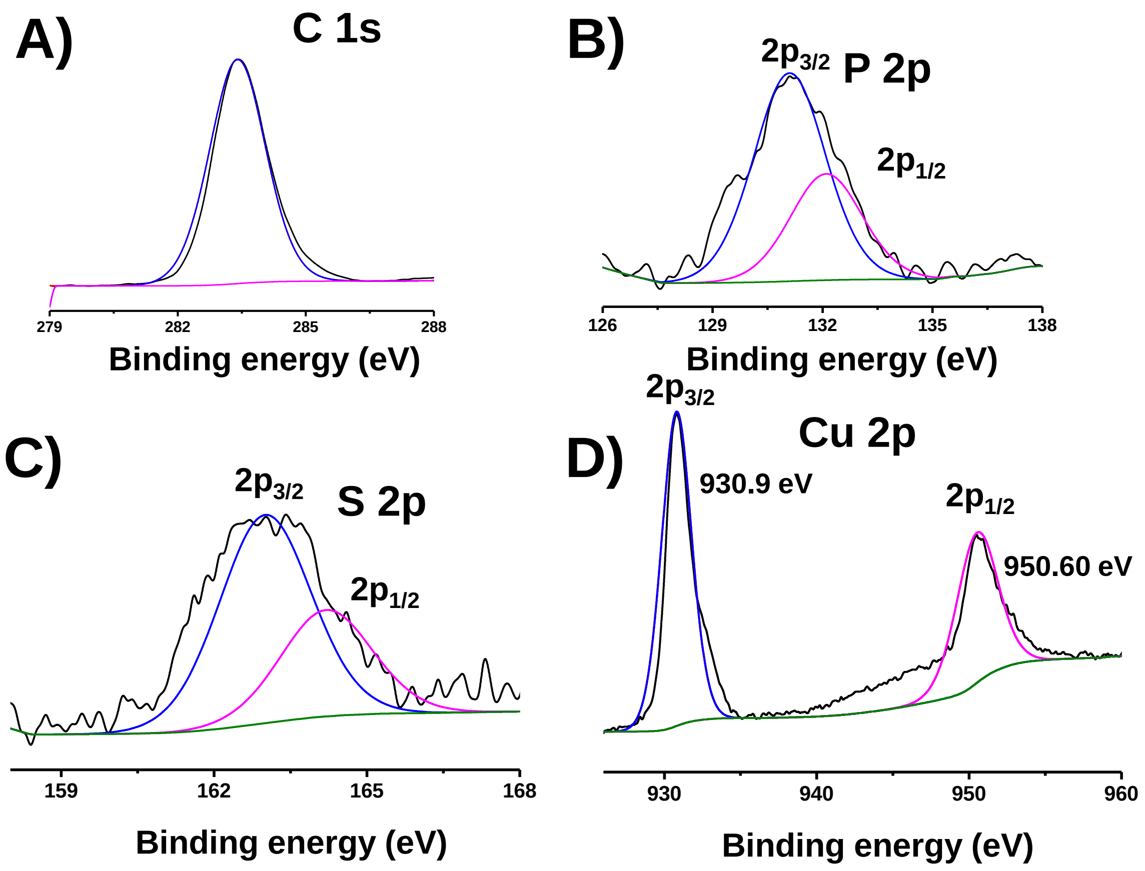

Figure S3. High-resolution XPS spectra of Cu_{15} NC; A) C 1s, B) P 2p, C) S 2p, and D) Cu 2p.

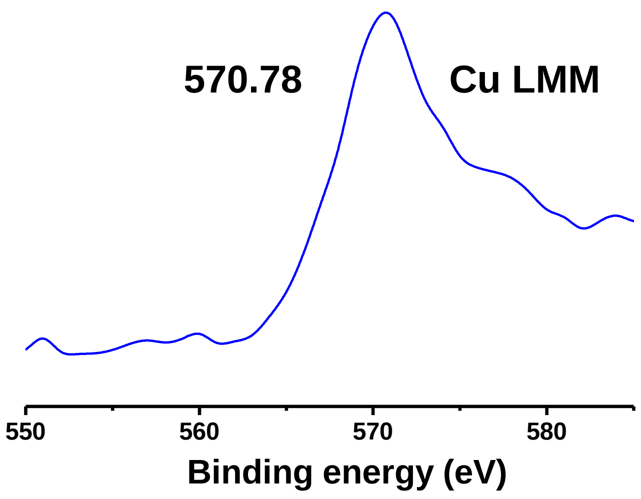

Figure S4. Cu LMM auger spectrum of Cu_{15} NC.

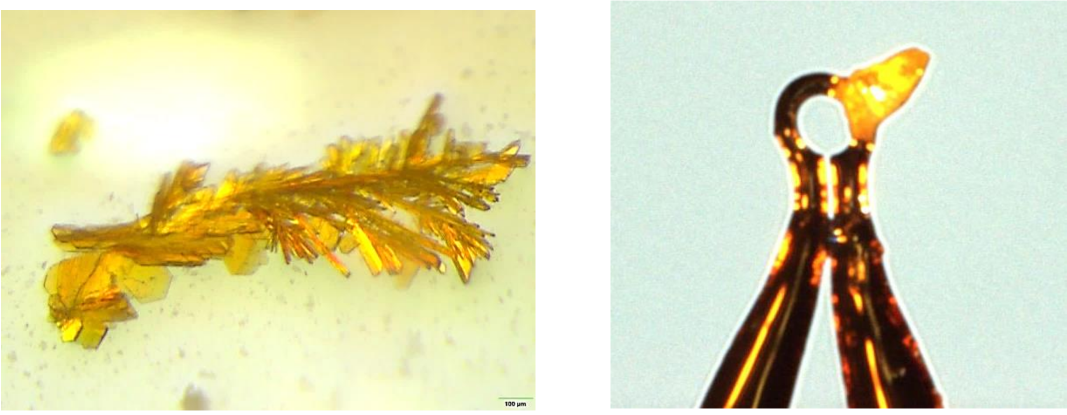

Figure S5. Images of Cu_{18} NC crystals.

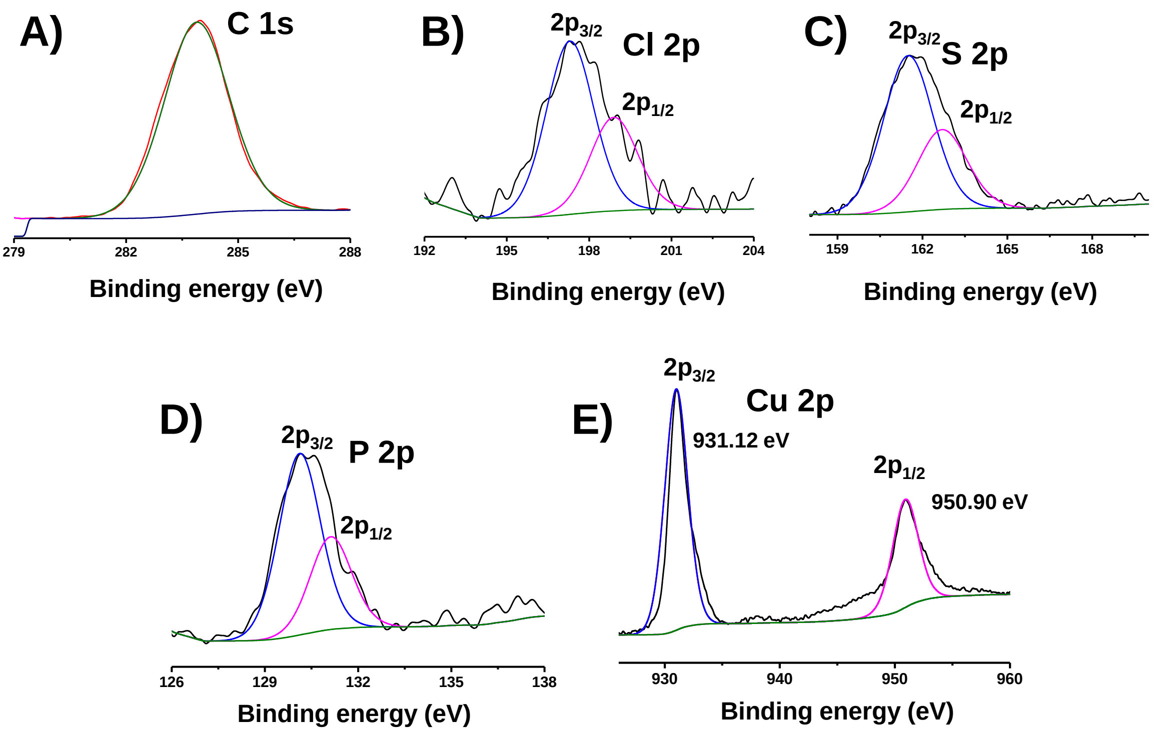

Figure S6. High-resolution XPS spectra of Cu_{15} NC; A) C 1s, B) Cl 2p, C) S 2p, D) P 2p, and E) Cu 2p.

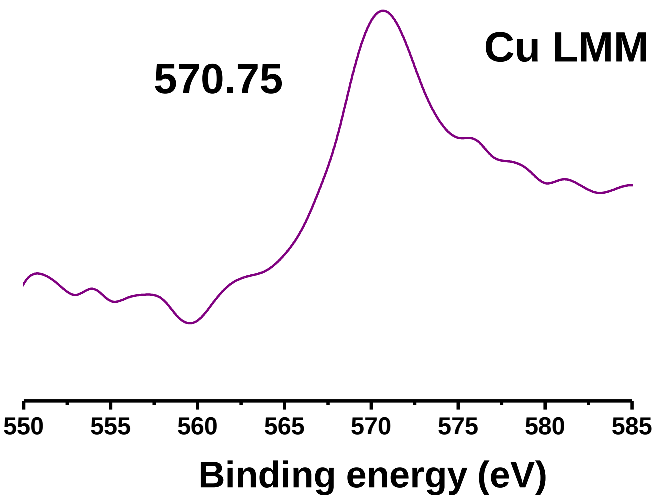

Figure S7. Cu LMM auger spectrum of Cu_{18} NC.

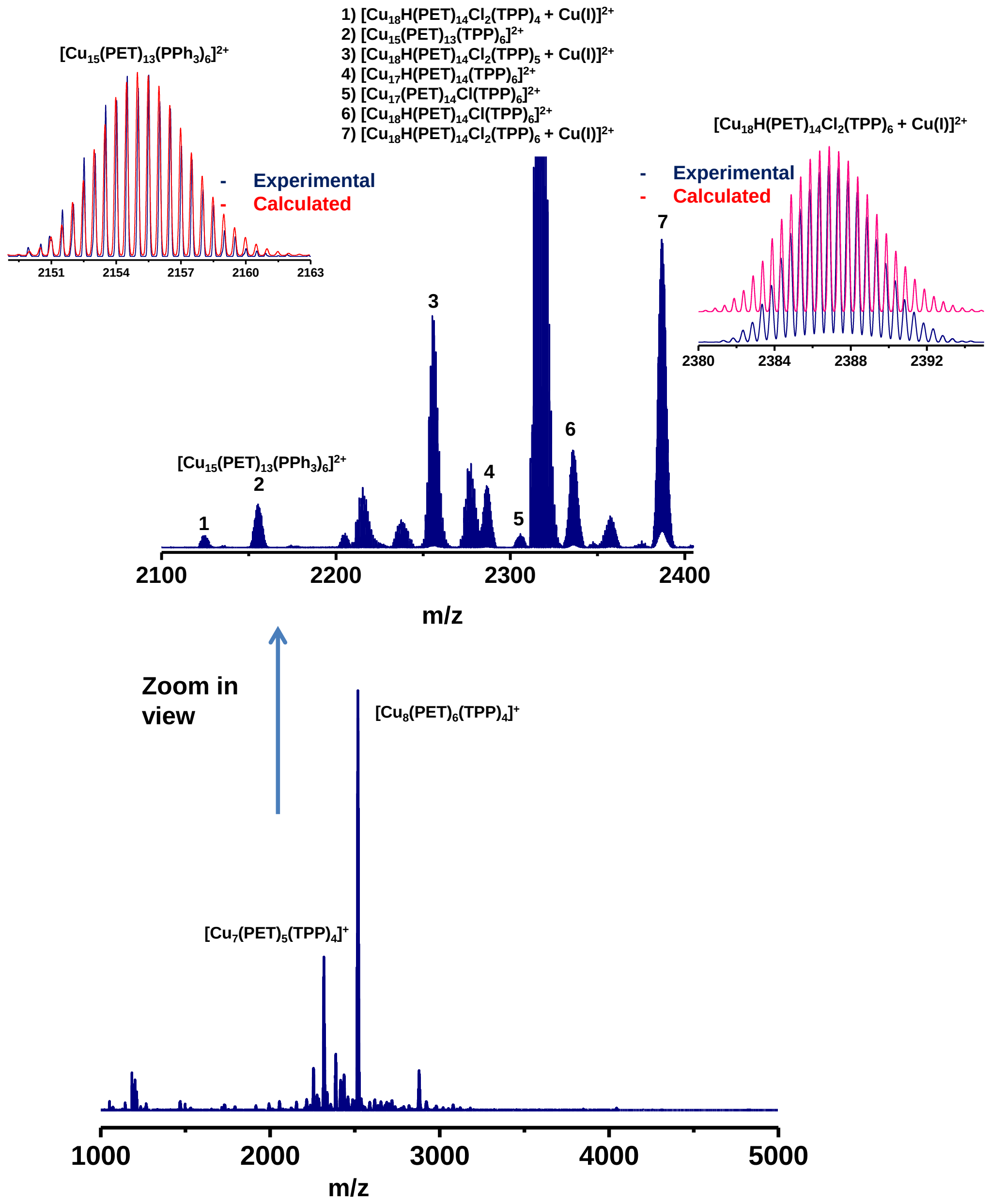

Figure S8. HR-ESI-MS of Cu_{15} and Cu_{18} NC in positive-ion mode after dissolving the precipitate in CH_2Cl_2. Comparison of experimental mass spectra of $[Cu_{15}(PET)_{13}(TPP)_6]^{2+}$ and $[Cu_{18}H(PET)_{14}Cl_2(TPP)_8 + Cu(I)]^{2+}$ with the simulated is provided in the inset of Figure S7. $[Cu_8(PET)_6(TPP)_4]^+$ and $[Cu_7(PET)_5(TPP)_4]^+$ are single charged species coming from the fragmentation of the NC under mass spec source condition.[12]

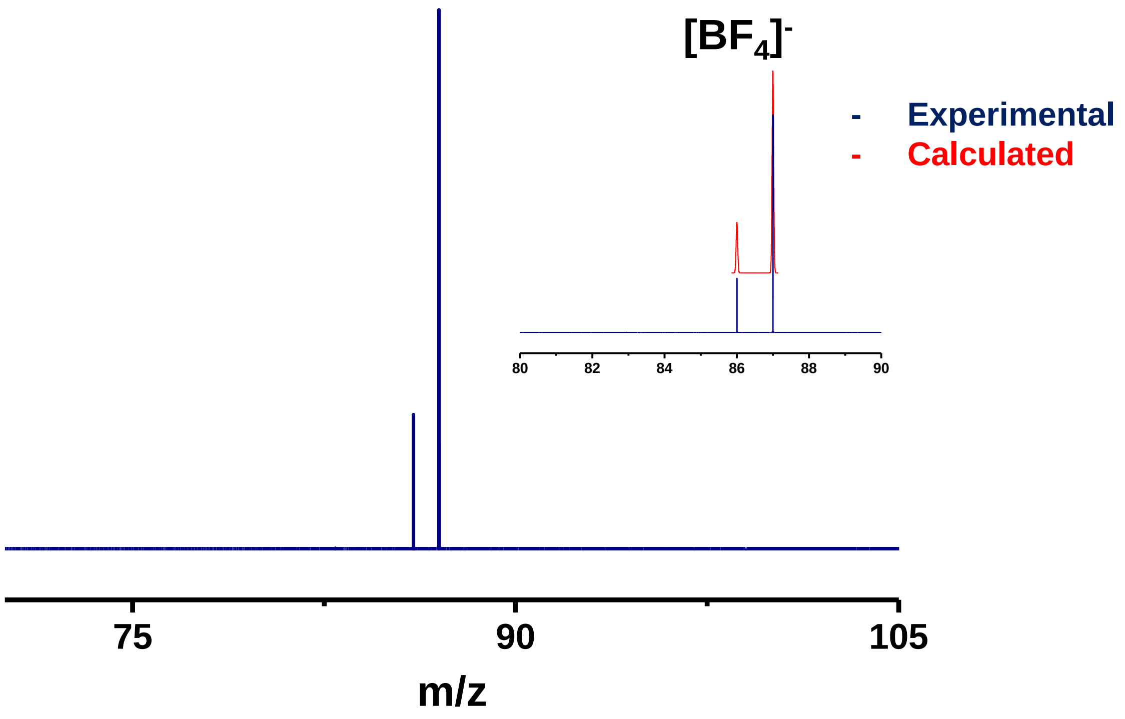

Figure S9. ESI-MS spectra of BF_4^- in negative mode after dissolving the crystals in DCM. Comparison of experimental mass spectra of BF_4^- with the simulated is provided in the inset of Figure S7.

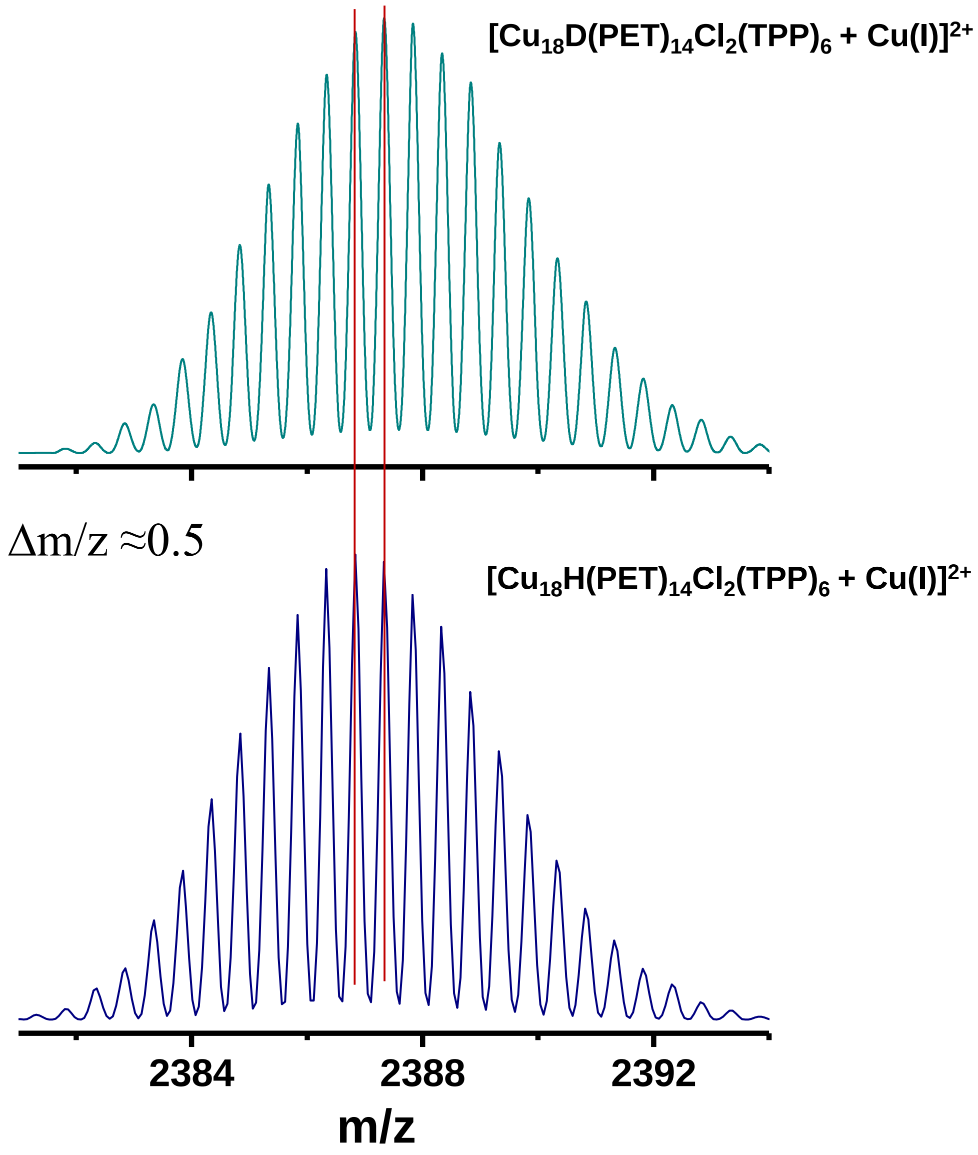

Figure S10. Comparison of the ESI-MS spectra of the Cu_{18} NC with the deuterated Cu_{18} NC in positive mode.

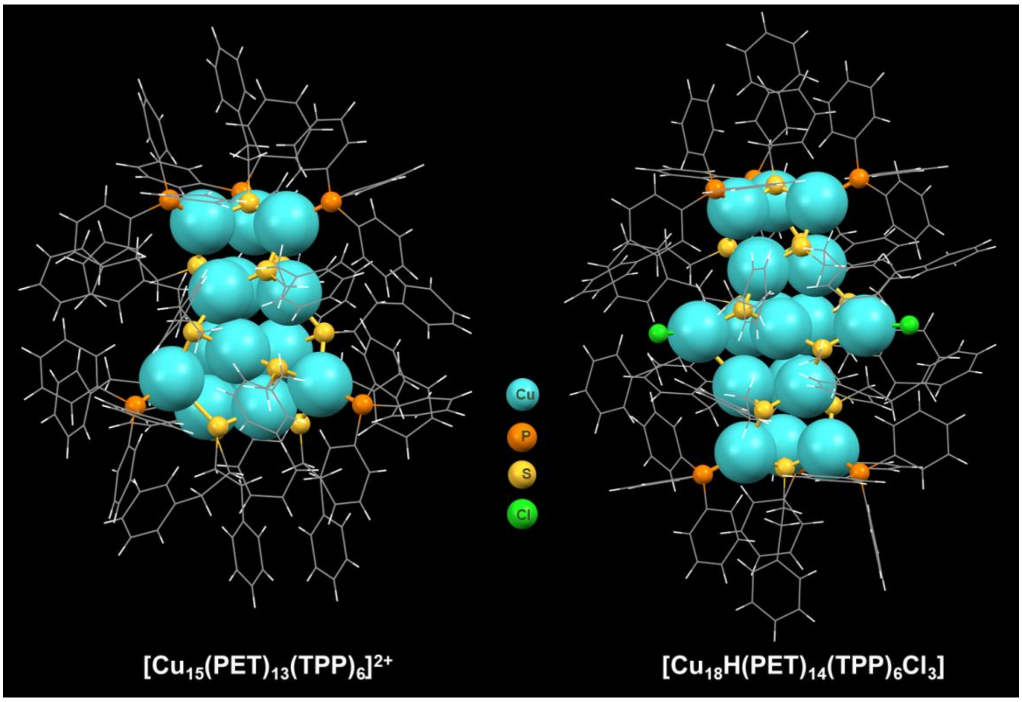

Figure S11. Comparison of the structures of Cu_{15} and Cu_{18} NC showing similar chiral triple-stranded helicate metal core.

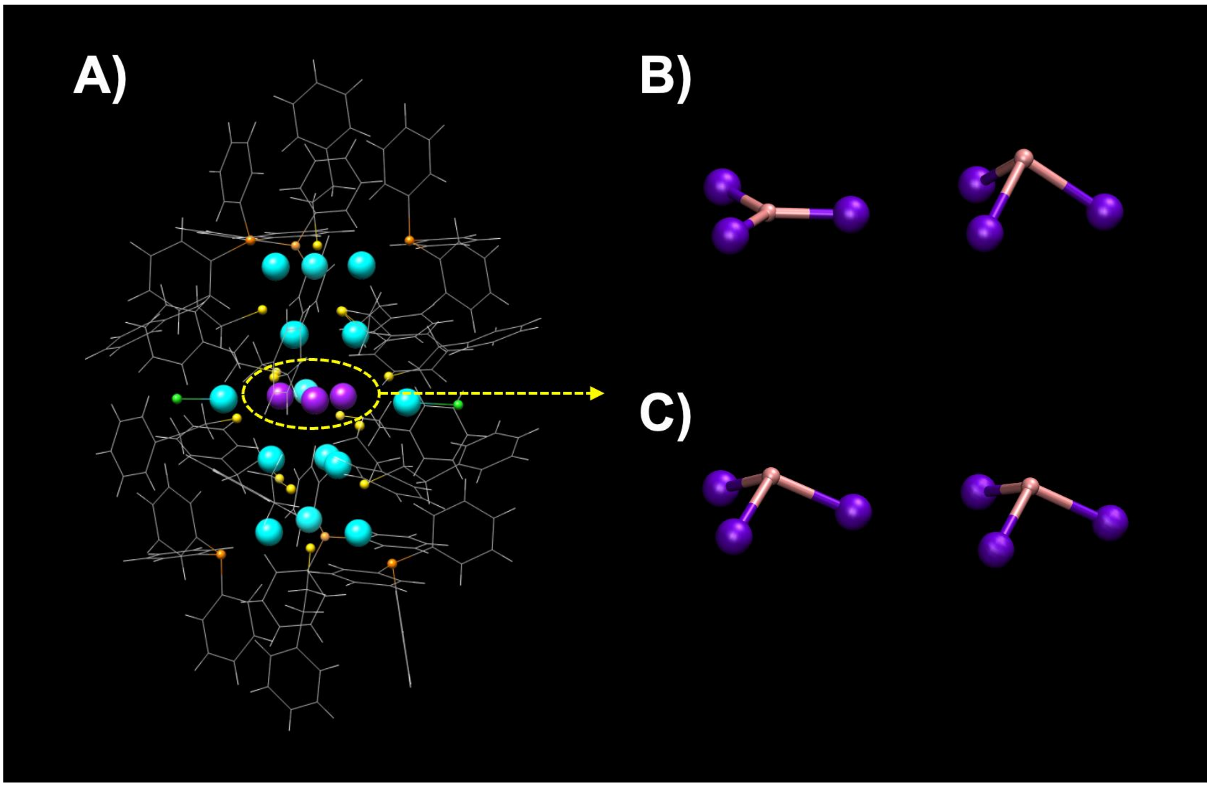

Figure S12. (A) The model structure of Cu_{18} NC used for DFT calculations. The Cu atoms are represented in cyan, with the three nearest Cu atoms to the hydride are highlighted in purple. (B) The three nearest Cu atoms and the hydride (pink) are shown before optimization and (C) after optimization, where nearly identical hydride positions were obtained for both the planar (left) and pyramidal (right) geometries.

**Table S1 Crystal data and structure refinement for Cu_{15} NC**	
Identification code	Cu15
CCDC No.	2345183
Empirical formula	$C_{218.84}H_{219.6}B_2Cl_6Cu_{15}F_8N_{1.75}O_{1.34}P_6S_{13}$
Formula weight	4837.59
Temperature/K	99.8(4)
Crystal system	triclinic
Space group	*P*-1
a/Å	18.8140(3)
b/Å	32.3256(5)
c/Å	38.2569(9)
α/°	112.773(2)
β/°	102.902(2)
γ/°	90.4260(10)
Volume/Å^3	20797.1(7)
Z	4
ρ_{calc}g/cm^3	1.545
μ/mm^{-1}	4.462
F(000)	9890.0
Crystal size/mm^3	0.26 × 0.18 × 0.04
Radiation	Cu Kα (λ = 1.54184)
2Θ range for data collection/°	4.66 to 134.682
Index ranges	$-22 \leq h \leq 21$, $-38 \leq k \leq 35$, $0 \leq l \leq 45$
Reflections collected	73536
Independent reflections	73536 [R_{int} = ?, R_{sigma} = 0.0796]
Data/restraints/parameters	73536/2703/4262
Goodness-of-fit on F^2	2.415
Final R indexes [I>=2σ (I)]	R_1 = 0.2188, wR_2 = 0.5604
Final R indexes [all data]	R_1 = 0.2520, wR_2 = 0.5806
Largest diff. peak/hole / e Å^{-3}	2.62/-1.57

Table S2. Crystal data and structure refinement for Cu_{18} NC

Identification code	Cu2202
CCDC No.	2345180
Empirical formula	$C_{220}H_{216}Cl_3Cu_{18}P_6S_{14}$
Formula weight	5017.98
Temperature/K	99.8(5)
Crystal system	Monoclinic
Space group	*C*2/*c*
a/Å	25.9002(3)
b/Å	27.6131(7)
c/Å	32.6918(10)
α/°	90
β/°	97.250(2)
γ/°	90
Volume/Å^3	23193.7(10)
Z	4
ρ_{calc}g/cm^3	1.437
μ/mm^{-1}	4.021
F(000)	10292.0
Crystal size/mm^3	0.17 × 0.103 × 0.083
Radiation	Cu Kα (λ = 1.54184)
2Θ range for data collection/°	6.86 to 133.178
Index ranges	$-22 \leq h \leq 30$, $-32 \leq k \leq 32$, $-38 \leq l \leq 38$
Reflections collected	113404
Independent reflections	20281 [R_{int} = 0.0862, R_{sigma} = 0.0550]
Data/restraints/parameters	20281/464/985
Goodness-of-fit on F^2	1.176
Final R indexes [I>=2σ (I)]	R_1 = 0.1063, wR_2 = 0.3238
Final R indexes [all data]	R_1 = 0.1645, wR_2 = 0.3781
Largest diff. peak/hole / e Å^{-3}	0.75/-0.54

Table S3. Comparison of the unit cell parameters between the previously reported Cu_{18}[12] and the present work

Unit cell parameters	This work	Reported[12]
Length (Å)	a = 25.9002(3)<br>b = 27.6131(7)<br>c = 32.6918(10)	a = 29.6954(15)<br>b = 24.3247(14)<br>c = 32.6835(18)
Angle (°)	α = 90<br>β = 97.250(2)<br>γ = 90	α = 90<br>β = 107.053(6)<br>γ = 90
Volume ($Å^3$)	23193.7(10)	22570(2)
Z	4	4
Density (g/cm^3)	1.437	1.416

References

1. C. Dong, R.-W. Huang, C. Chen, J. Chen, S. Nematulloev, X. Guo, A. Ghosh, B. Alamer, N. M. Hedhili, T. T. Isimjan, et al., *J. Am. Chem. Soc.* 2021, *143*, 11026-11035.
2. O. V. Dolomanov, L. J. Bourhis, R. J. Gildea, J. A. K. Howard, H. Puschmann, J. Appl. Crystallogr., 2009, 42, 339-341.
3. G. M. Sheldrick, *Acta Crystallogr. A: Found. Adv.,* 2015, 71, 3-8.
4. G. M. Sheldrick, Acta crystallogr., C Struct. chem., 2015, 71, 3-8.
5. G. M. Sheldrick, Acta crystallogr., C Struct. chem., 2015, 71, 3-8.
6. L. J. Farrugia, Journal of Appled Crystallography 1997, 30, 565–565.
7. S. Nematulloev, R.-W. Huang, J. Yin, A. Shkurenko, C. Dong, A. Ghosh, B. Alamer, R. Naphade, M. N. Hedhili, P. Maity, M. Eddaoudi, O. F. Mohammed, O. M. Bakr, *Small* 2021, 17, 2006839.
8. M. J. Frisch, G. W. Trucks, H. B. Schlegel, G. E. Scuseria, M. A. Robb, J. R. Cheeseman, G. Scalmani, V. Barone, G. A. Petersson, H. Nakatsuji, X. Li, M. Caricato, A. V. Marenich, J. Bloino, B. G. Janesko, R. Gomperts, B. Mennucci, H. P. Hratchian, J. V. Ortiz, A. F. Izmaylov, J. L. Sonnenberg, D. Williams-Young, F. Ding, F. Lipparini, F. Egidi, J. Goings, B.

Peng, A. Petrone, T. Henderson, D. Ranasinghe, V. G. Zakrzewski, J. Gao, N. Rega, G. Zheng, W. Liang, M. Hada, M. Ehara, K. Toyota, R. Fukuda, J. Hasegawa, M. Ishida, T. Nakajima, Y. Honda, O. Kitao, H. Nakai, T. Vreven, K. Throssell, J. A. Montgomery, Jr., J. E. Peralta, F. Ogliaro, M. J. Bearpark, J. J. Heyd, E. N. Brothers, K. N. Kudin, V. N. Staroverov, T. A. Keith, R. Kobayashi, J. Normand, K. Raghavachari, A. P. Rendell, J. C. Burant, S. S. Iyengar, J. Tomasi, M. Cossi, J. M. Millam, M. Klene, C. Adamo, R. Cammi, J. W. Ochterski, R. L. Martin, K. Morokuma, O. Farkas, J. B. Foresman, and D. J. Fox, Gaussian, Inc., Wallingford CT, 2016.

9. A. D. Becke, J. Chem. Phys., 1993, 98, 5648-5652.
10. S. Grimme, S. Ehrlich, L. Goerigk, J. Comput. Chem. 2011, 32, 1456-1465.
11. C. Lee, W. Yang, R.G. Parr, Physical Review B 1988, 37, 785-789.
12. G. Dong, Z. Pan, B. Han, Y. Tao, X. Chen, G. G. Luo, P. Sun, C. Sun, D. Sun, *Angew. Chem. Int. Ed.* 2023, *62*, e202302595.
